# Achievement of recommended targets for cardiovascular disease prevention in adults with diabetes in 38 low- and middle-income countries

**DOI:** 10.7189/jogh.14.04148

**Published:** 2024-09-20

**Authors:** Yang Li, Minghai Yan, Qiujing Cai, Lap Ah Tse, Zhiguang Liu, Xinyue Lang, Biyan Wang, Qiuyan Ma, Mengya Li, Qi Qiu, Wei Li

**Affiliations:** 1 Interventional Centre of Valvular Heart Diseases, Beijing Anzhen Hospital, Capital Medical University, Beijing, China; 2 Medical Research and Biometrics Centre, National Clinical Research Centre for Cardiovascular Diseases, Fuwai Hospital, National Centre for Cardiovascular Diseases, Chinese Academy of Medical Sciences and Peking Union Medical College, Beijing, China; 3 Jockey Club School of Public Health and Primary Care, Faculty of Medicine, Chinese University of Hong Kong, Hong Kong, China; 4 Clinical Trial Unit, Beijing Anzhen Hospital, Capital Medical University, Beijing, China

## Abstract

**Background:**

Implementation of guideline recommendations for cardiovascular disease (CVD) prevention in people with diabetes in low- and middle-income countries (LMICs) is unclear. We assessed the achievement of CVD prevention targets among patients with diabetes in LMICs.

**Methods:**

We pooled nationally representative cross-sectional surveys from 38 LMICs. We evaluated three targets according to the World Health Organization’s (WHO) recommendations: treatment (glucose-lowering drugs, statins, antihypertensive drugs, and aspirin); metabolism (blood glucose, body mass index, blood pressure, and cholesterol); and lifestyle (non-smoking, non-drinking, physical activity, and diet). We used multivariable Poisson regression models to assess sociodemographic factors influencing adherence to guideline recommendations.

**Results:**

The study included 110 083 participants, of whom 6789 (6.0%) had self-reported diabetes. The prevalence of achieving the treatment, metabolic and lifestyle targets for all components were 9.9%, 8.1%, and 7.2%, respectively. The components with the lowest prevalence of the three targets were 11.1% for statin use, 27.3% for body mass index control, and 19.5% for sufficient consumption of fruit and vegetables, respectively. Upper-middle-income countries were better at achieving the treatment, non-drinking, and dietary targets than lower-middle-income countries. Women, middle-aged and older patients, and highly educated patients had a lower prevalence of metabolic adherence.

**Conclusions:**

In LMICs, the prevalence of patients with diabetes meeting WHO-recommended treatment, metabolic and lifestyle targets for CVD prevention was low. Our findings highlighted the need to strengthen the prevention of CVD in patients with diabetes in LMICs.

Diabetes is currently the eighth leading cause of death and disability worldwide [1]. By 2021, the total number of people with diabetes worldwide had reached 529 million, 80% of whom lived in low- and middle-income countries (LMICs) [2]. In addition, the prevalence of diabetes and the number of people affected are growing faster in LMICs compared to high-income countries [3]. People with diabetes in LMICs also have higher rates of cardiovascular disease (CVD) morbidity and mortality [4].

Diabetes increases the risk of many types of CVD, particularly ischemic heart disease and stroke, and advances the age of onset of CVD [5-7]. At the same time, conditions that often cluster with diabetes, such as obesity, hypertension and dyslipidaemia, are also risk factors for CVD [8]. Pharmacological treatment to control cardiometabolic risk factors such as blood glucose, blood pressure and lipids has long been a common recommendation in guidelines for the prevention of CVD in people with diabetes worldwide [9-11]. In addition, adopting healthy lifestyles such as weight control, a healthy diet, and sufficient physical activity have been shown to play an important role in non-pharmacological interventions to reduce the incidence of CVD in people with diabetes [12]. Unfortunately, despite our recognition of the positive role of personalised diabetes management in the development of CVD, the achievement of guideline-recommended targets is poor worldwide, and there is no trend towards improvement [13]. In LMICs, disadvantageous social determinants of health, inadequate levels of universal health coverage, and poor accessibility and availability of diabetes-related health services have undoubtedly fuelled the rapid rise in diabetic CVD [14,15]. Previous studies have reported on the achievement of some guideline-recommended targets, such as glucose-lowering drugs, antihypertensive drugs and statins, or glycaemic and blood pressure control, in patients with diabetes in LMICs. The study results were as expected, with a large unmet need for diabetes treatment and control in LMICs and large regional heterogeneity [16,17]. However, strategies to reduce the risk of CVD in patients with diabetes should be comprehensive, based on a healthy lifestyle and supported by pharmacological management to optimise lipid levels and control blood pressure and blood glucose [18]. However, to the best of our knowledge, there is a lack of studies that comprehensively assess the current status of CVD prevention in people with diabetes in LMICs from a therapeutic, metabolic, and lifestyle perspective.

As CVD prevention in diabetes is also an implementation priority of the Global Diabetes Compact, assessing the current status of prevention efforts in LMICs can identify gaps between the current state of health care implementation and the desired targets, thus contributing to the global goal of a 30% reduction in premature deaths from chronic noncommunicable diseases by 2030 [14,19]. In this study, we aimed to assess the prevalence of meeting current guideline-recommended CVD treatment, metabolic, and lifestyle targets among people with diabetes in 38 LMICs and to explore differences in target achievement at the level of national and individual characteristics.

## METHODS

### Study design and data source

We pooled nationally representative Stepwise Approach to Surveillance (STEPS) surveys conducted by the World Health Organization (WHO) in 38 LMICs between 2013–20. This cross-sectional observational study assessed metabolic, behavioural, and psychosocial risk factors for chronic non-communicable diseases, such as cardiovascular disease and diabetes, as well as participants’ use of health care services. Implementation details and coordination methods have been published previously [20], and key details are presented in Appendix S1 in the **Online Supplementary Document**. Countries are categorised into World Bank income groups and six geographic regions (Appendix S1–2 in the **Online Supplementary Document**). 2013 was chosen as the starting year because STEPS introduced the question of personal CVD history. Ethical approval for our study was waived by the Fu Wai Hospital ethics committee due to open access and the inability to link to individual participant data.

The population consisted of adults aged 25–69 years. The age selection was consistent with the age range of most national surveys. We included and excluded national individual databased on a number of criteria (Appendix S1 in the **Online Supplementary Document**).

### Outcomes and procedures

The primary outcomes were the prevalence of meeting current guideline-recommended individualised treatment (glucose-lowering medications, antihypertensive medications, statins, and aspirin), metabolic (blood glucose, body mass index (BMI), blood pressure, and total cholesterol), and healthy lifestyle (non-smoking, non-drinking, adequate physical activity, and a rational diet) targets for CVD prevention in people with self-reported diabetes. Self-reported diabetes was defined as a self-reported physician-diagnosed diabetes or use of glucose-lowering medication. The targets were set with primary reference to the WHO guidelines for preventing CVD [21]. In this guideline, the management of patients with diabetes is primarily based on the 12 sub-goals listed above. Ideally, each patient should achieve all of the individual targets.

Medication use was assessed using a standardised individual questionnaire. To maintain consistency with guideline recommendations, antihypertensive medication use was assessed in patients with hypertension. Statin use was assessed in patients aged ≥40 years. Aspirin use was assessed in people with diabetes with a 10-year CVD risk >20% or a history of CVD. Hypertension was defined as the average of three or two blood pressure measurements ≥140/90 mm Hg or the use of antihypertensive medication. The 10-year risk of CVD was calculated using the WHO’s CVD risk laboratory-based chart [22]. CVD history was self-reported by answering the question ‘Have you ever had a heart attack or chest pain from heart disease (angina) or a stroke (cerebrovascular accident or incident)?’

Venous blood was collected for fasting blood glucose and total cholesterol after an overnight fast of at least eight hours. Participants took three or two blood pressure measurements after a break of at least five minutes in the field, and the average of the measurements was used to represent the final systolic and diastolic blood pressures. The fasting blood glucose target was <6.1 mmol/L (110 mg/dL). BMI targets were set at <25 kg/m^2^ and 23 kg/m^2^ for South-East Asians. The blood pressure target was set at <140/90 mm Hg. Total cholesterol targets were set at <5.0 mmol/L (190 mg/dL) for patients without a history of CVD and <4.0 mmol/L (152 mg/dL) for patients with a history of CVD.

A healthy lifestyle was assessed by self-report using questionnaires. Current non-smoking was defined as not using tobacco products in the past year. Current non-drinking was defined as not drinking alcohol in the past 30 days. Adequate physical activity was defined as ≥150 minutes of moderate-intensity activity at work or leisure time or ≥75 minutes of vigorous-intensity activity in a typical week in the past year. A rational diet was defined as an average daily intake of ≥5 servings of fruit or vegetables in a typical week in the past year.

### Statistical analyses

Given the design characteristics of the STEPS multistage stratified random sample, we included pre-calculated weights from the survey team. We used the Stata command ‘svyset’ to correct for stratification and clustering effects in the primary sampling units, allowing for a robust error structure in the analysis. Sample weights were used to account for population, selection, and non-response errors and to minimise differences between the sample and the total. We adjusted the weights of the combined and relevant subgroup samples for the 38 countries to give each country the same weight, and the country level was treated as a fixed effect. This has the advantage that the contribution of each survey is equalised, and the pooled results are not dominated by surveys from countries with large populations.

First, we reported the overall prevalence of meeting therapeutic (glucose-lowering medications, antihypertensive medications, statins, and aspirin), metabolic (glucose, BMI, blood pressure, and total cholesterol), and healthy lifestyle (smoking cessation, alcohol restriction, adequate physical activity, and rational diet) targets for CVD prevention in people with self-reported diabetes, and separately by World Bank income groups, WHO regions, and countries. Second, to reflect the combined level of CVD prevention in diabetes, we estimated the composite prevalence of four treatments, four metabolic, and four lifestyle targets. In addition, to explore individual characteristics associated with better target attainment in the pooled sample, we reported the prevalence for subgroups of age (25–39, 40–54, and 55–69 years), sex, and educational attainment (no formal schooling, primary, and secondary or higher) and fitted multivariable Poisson regression models, including country level as a fixed effect and estimating the absolute difference in prevalence between groups in terms of target attainment using the average marginal effect model.

We conducted sensitivity analyses to demonstrate the robustness of the results. First, the weights were reweighted using the proportion of the population aged 25–69 years in 38 countries. Second, the blood pressure target was set at <130/80 mm Hg. Finally, the 2007 WHO International Society of Hypertension CVD risk charts were used to calculate the 10-year CVD risk of >30% as the target population for aspirin primary prevention [21]. We conducted all analyses with Stata, version 18.0 (StataCorp LLC, College Station, Texas, USA) and R, version 4.2.2 (R Core Team, Vienna, Austria). (Appendix S1 in the Online **Supplementary Document**).

## RESULTS

### Survey characteristics

The pooled sample consisted of 6789 self-reported diabetes patients aged 25–69 years with a median age of 54 years (interquartile range (IQR) = 45–61) from 38 LMICs, of whom 4310 were female (54.8%; 95% confidence interval (CI) = 52.5–57.0) and 2479 were male (45.2%; 95% CI = 43.0–47.5). By WHO region, the pooled sample includes two countries in the Americas, five in South-East Asia, six in the Eastern Mediterranean, six in the Western Pacific, eight in Europe, and 11 in Africa. By World Bank income group, the sample includes nine low-income countries18, lower-middle-income countries, and 11 upper-middle-income countries. The median response rate for all surveys was 87% (IQR = 74–95) (**Table 1**, Appendix S2 in the **Online Supplementary Document**).

**Table 1:** Individual characteristics of the pooled sample and people with diabetes*

Characteristics	Total population (n = 110 083)		Diabetes (n = 6789)	
**Unweighted (n)**	**Weighted (%)**	**Unweighted (n)**	**Weighted (%)**
Age in years				
*25–39*	45 003	48.7	878	18.2
*40–54*	39 266	32.7	2610	38.5
*55–69*	25 814	18.6	3301	43.3
Sex				
*Male*	43 982	48.9	2479	45.2
*Female*	66 982	51.1	4310	54.8
Country economic status				
*Upper-middle*	25 573	28.9	2260	28.9
*Lower-middle*	63 723	55.3	3992	55.3
*Low*	20 787	15.8	537	15.8
Region				
*Africa*	31 103	28.9	889	28.9
*Americas*	3994	5.3	367	5.3
*Western Pacific*	11 524	15.8	713	15.8
*European*	21 911	21.1	1398	21.1
*Eastern Mediterranean*	17 979	15.8	1853	15.8
*South-East Asia*	23 572	13.2	1569	13.2
Education				
*No schooling*	18 053	12.5	951	11.9
*Primary*	39 257	37.6	2493	38.1
*Secondary or higher*	48 697	49.9	3128	50.0
Area of residence				
*Urban*	32 981	42.3	2599	56.2
*Rural*	42 046	57.7	1607	43.8

*Prevalence was calculated based on the adjusted weight, giving equal weight to each country.

### Treatment, metabolic and lifestyle targets for CVD prevention in people with diabetes

**Figure 1:**
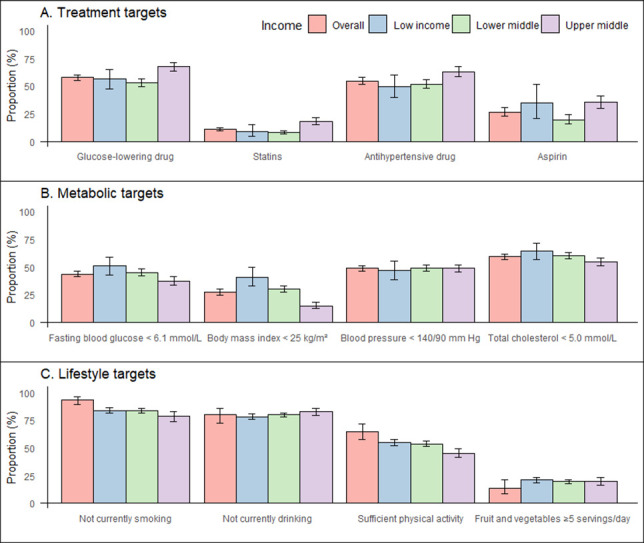
Prevalence of treatment, metabolic and lifestyle targets for CVD prevention in patients with diabetes. Sample weights have been adjusted to give equal weight to each country. Error bars represent 95% confidence intervals. The prevalence of statin use was assessed in people with diabetes aged ≥40 years. The prevalence of aspirin use was assessed in people with diabetes with a history of CVD and a 10-year risk of CVD>20%, as calculated using the WHO’s CVD risk laboratory-based chart. Bangladesh, Myanmar, Nepal, Sir Lanka, and Timor-Leste have a body mass index target of <23 kg/m^2^. Total cholesterol targets have been set at <5.0 mmol/L (190 mg/dL) for patients without a history of CVD and <4.0 mmol/L (152 mg/dL) for patients with a history of CVD. CVD – cardiovascular disease.

**Figure 2:**
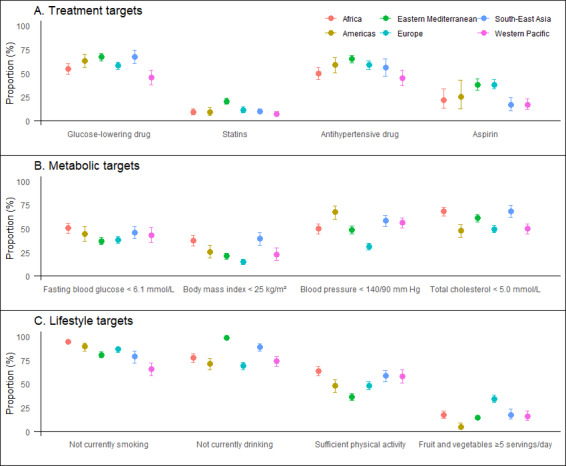
Prevalence of treatment, metabolic and lifestyle targets for CVD prevention in patients with diabetes by region. Sample weights have been adjusted to give equal weight to each country. Error bars represent 95% confidence intervals. The prevalence of statin use was assessed in people with diabetes aged ≥40 years. The prevalence of aspirin use was assessed in people with diabetes with a history of CVD and a 10-year risk of CVD>20%, as calculated using the WHO’s CVD Risk Laboratory-based Chart. Bangladesh, Myanmar, Nepal, Sir Lanka, and Timor-Leste have a body mass index target of <23 kg/m^2^. Total cholesterol targets have been set at <5.0 mmol/L (190 mg/dL) for patients without a history of CVD and <4.0 mmol/L (152 mg/dL) for patients with a history of CVD. CVD – cardiovascular disease.

Regarding treatment targets, 57.9% (95% CI = 55.4–60.3) of people with diabetes took glucose-lowering drugs. 11.1% (95% CI = 9.9–12.6) of patients aged >40 years were using statins. Moreover, 54.7% (95% CI = 51.8–57.6) of patients with hypertension were taking antihypertensive medication. Further, 26.5% (95% CI = 22.9–30.5) of people at high risk of CVD were taking aspirin. Regarding WHO regional distribution, the Eastern Mediterranean region achieved the four treatment targets better than the other regions, and the Western Pacific region achieved the worst (**Figure 1**, **Figure 2**, Appendix S3 in the **Online Supplementary Document**).

For the metabolic targets, 43.7% (95% CI = 41.2–46.2) of people with diabetes had good glycaemic control. 27.3% (95% CI = 24.8–29.8) had good weight control, and 48.7% (95% CI = 46.5–50.9%) had good blood pressure control. 59.2% (95% CI = 56.9–61.5) had good control of total cholesterol. At the regional level, Africa and South-East Asia performed better, while the European region performed worse.

Regarding lifestyle targets, 83.8% (95% CI = 81.6–85.9) were current non-smokers. 79.4% (95% CI = 77.9–81.7) were current non-drinkers. 53.7% (95% CI = 51.4–56.0) are physically active. 19.5% (95% CI = 17.7–21.5) consumed ≥5 portions of fruit or vegetables daily. At the regional level, the Western Pacific performed poorly on the non-smoking target, Europe on the non-alcohol target, the Eastern Mediterranean on the physical activity target, and the Americas on the dietary target (Appendix S3 in the **Online Supplementary Document**).

### Combination of treatment, metabolic and lifestyle targets in the pooled sample

**Figure 3:**
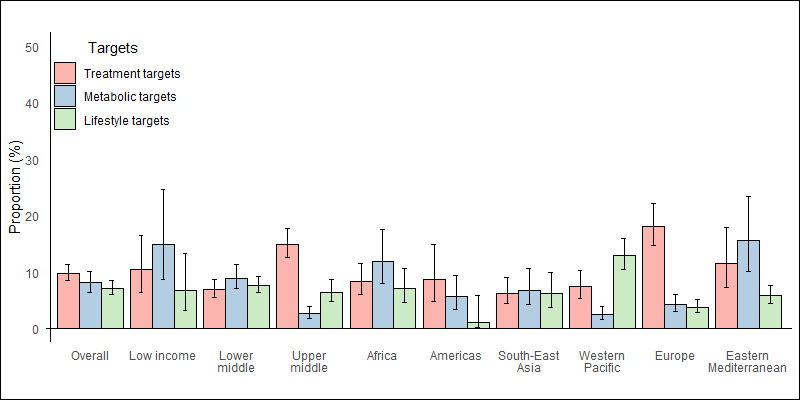
Prevalence of guideline-recommended targets for all treatment, metabolic and lifestyle components of CVD prevention in patients with diabetes, by World Bank income group and region. Sample weights have been adjusted to give equal weight to each country. Error bars represent 95% confidence intervals. CVD – cardiovascular disease.

Overall, the prevalence of achieving the treatment, metabolic, and lifestyle targets for all components was 9.9% (95% CI = 8.5–11.3), 8.1% (95% CI = 6.5–10.1) and 7.2% (95% CI = 6.1–8.5), respectively. Looking at the World Bank income group, upper-middle-income countries had the best achievement of treatment targets but the worst achievement of metabolic targets, with countries at different income levels having similar achievements of the combined lifestyle targets. Looking at the WHO regions, the Eastern Mediterranean had better compliance with treatment targets, South-East Asia with metabolic targets and Europe with lifestyle targets (**Figure 3**, Appendix S4 in the **Online Supplementary Document**).

### Treatment, metabolic and lifestyle targets for CVD prevention across individual characteristics

**Figure 4:**
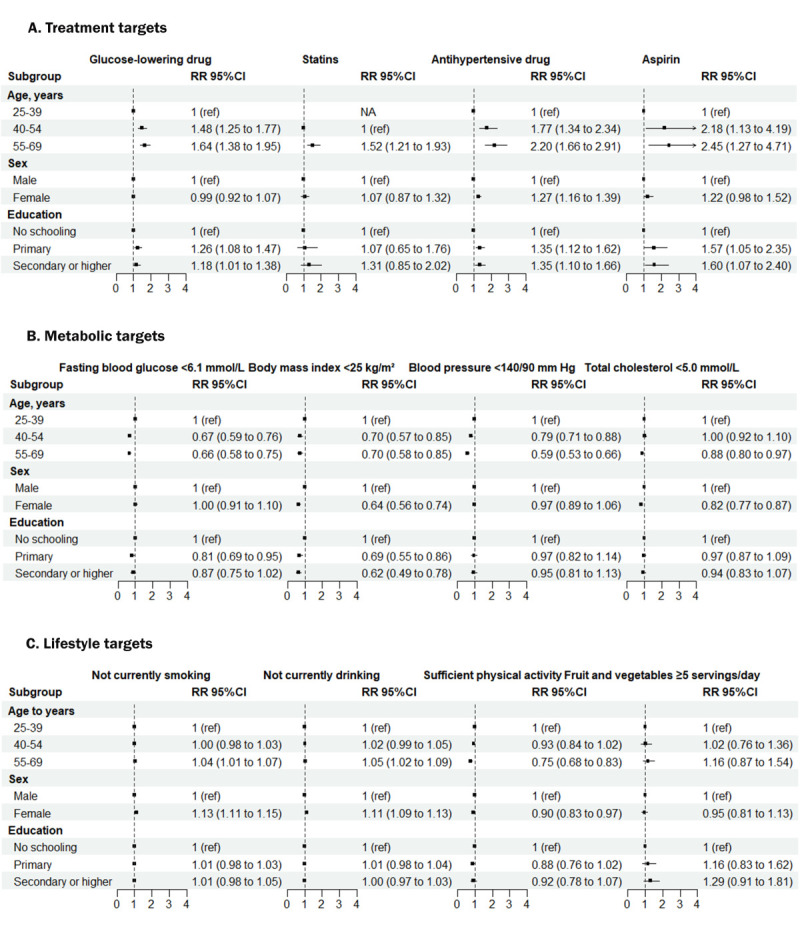
Association of individual characteristics with guideline-recommended treatment, metabolic and lifestyle targets in people with diabetes. Sample weights have been adjusted to give equal weight to each country. Error bars represent 95% confidence intervals. The multivariable Poisson regression models have been adjusted for age group, sex and education, including country-level fixed effects.

Overall, older age was associated with better achievement of treatment targets but worse achievement of metabolic targets. Women had higher BMI, total cholesterol control, and physical activity levels than men but were more likely to achieve non-smoking and non-alcohol targets. Higher levels of education were associated with a greater likelihood of achieving treatment targets but poorer BMI control (**Figure 4**, Appendix 5 in the **Online Supplementary Document**).

### Sensitivity analyses

After reweighting using each country’s population aged 25–69 years, the prevalence of meeting treatment, metabolic, and lifestyle targets did not change significantly, and the pattern of change between different income countries and regions was consistent with the main analysis. Using the WHO International Society of Hypertension risk charts to delineate the 10-year CVD high-risk population, aspirin use was 35.6% (95% CI = 31.7–39.8). Only 26.4% (95% CI = 21.5–32.0) achieved their blood pressure goal when the cut-off point for blood pressure control was set at 130/80 mm Hg (Appendix S6 in the **Online Supplementary Document**).

## DISCUSSION

A cross-sectional study of 38 LMICs found significant gaps in achieving guideline-recommended CVD prevention targets in patients with self-reported diabetes. For treatment targets, two-thirds of patients used glucose-lowering and antihypertensive medications, one-third of high-risk individuals used aspirin, and one-tenth used statins. In terms of metabolic targets, less than half of the patients had better control of fasting blood glucose and blood pressure, and less than a third had good control of weight. Regarding healthy lifestyles, half of the patients met their physical activity targets, and one in five met their dietary targets. Less than one in 10 patients achieved all components of the treatment, metabolic and lifestyle targets. At the national level, treatment, non-drinking and dietary targets were better achieved in upper-middle-income countries than in lower-middle-income and low-income countries, while metabolic, non-smoking and physical activity targets were poorly achieved. At the individual level, older age and higher levels of education were associated with better adherence to treatment targets and poorer adherence to metabolic targets. Women were less likely than men to achieve BMI, total cholesterol and physical activity targets.

Medication is a key component of CVD prevention in people with diabetes. Our study highlights the underuse of medication in people with diabetes in LMICs. Compared with the results of previous STEPS, our study found a higher prevalence of glucose-lowering medications and a similar prevalence of antihypertensive medications and statins [17]. This may be related to the fact that we only studied patients with self-reported diabetes. In the USA, the prevalence of glucose-lowering medication, antihypertensive medication, and statin use in patients with diabetes was 82.7%, 73.8%, and 56.3%, respectively [23]. In China, the prevalence was 84.3%, 37.1%, and 19.5%, respectively [24]. In India, the prevalence was 89.2%, 29.9%, and 15.5%, respectively [25]. Our observation of a lower prevalence of glucose-lowering medication use may be related to the inclusion of low-income countries in the study, and the higher prevalence of antihypertensive medication use may be related to the assessment of antihypertensive medication use only in hypertensive patients. Indeed, improving treatment coverage for people with diabetes in LMICs is a long-term issue. On the one hand, LMICs, especially low-income countries, have poor availability and affordability of drugs for CVD prevention [26,27]. On the other hand, physicians in LMICs have demonstrated therapeutic inertia more clearly [28]. Primary care physicians lack sufficient therapeutic experience to prescribe the optimal dose at the optimal time.

Overall, the prevalence of metabolic target achievement in people with diabetes is poor worldwide [13]. In the USA, 66.8% of patients with diabetes achieved individualised haemoglobin A1c (HbA1c) targets, 70.4% had a blood pressure <140/90 mm Hg, and 55.7% had a non-high-density lipoprotein cholesterol level <130 mg/dL, with one in five patients achieving all three targets [23,29]. In China, 64.1% of patients with diabetes achieved individualised HbA1c targets, 22.2% had blood pressure <130/80 mm Hg, 59.7% had low-density lipoprotein cholesterol levels <100 mg/dL, and 32.2% had a BMI<24 kg/m^2^ [24]. In India, 36.3% of people with diabetes had HbA1c <7.0%, 48.8% had blood pressure <140/90 mm Hg, and 63.8% had total cholesterol <200 mg/dL, which is similar to our findings [25]. In addition, we found that the prevalence of meeting metabolic targets was lower in upper-middle-income countries than in low-income countries, which may be due to the ageing of the population. Differences in the extent to which diabetes prevention activities are implemented in the health systems of different countries and individuals’ access to health services may account for differences in metabolic target achievement between countries [30]. In the future, the management of blood glucose, blood pressure and BMI in patients with diabetes should be strengthened, with a focus on older patients and women, to promote the early achievement of the 80% target of the Global Diabetes Compact.

Lifestyle interventions should be the basis for stemming the diabetes epidemic and reducing the spread of CVD complications in LMICs. In this study, about 80% of people with diabetes did not smoke or drink alcohol, which is similar to the global prevalence levels of tobacco and alcohol [31,32]. At the same time, we found that people with diabetes had a lower prevalence of meeting physical activity and dietary targets than the global average [33,34]. This may suggest that the next focus of lifestyle interventions for CVD prevention will be to address low physical activity and poor dietary habits among people with diabetes in LMICs. Key issues to be addressed included the lack of opportunities for physical activity in LMICs, the inaccessibility and affordability of fruit and vegetables, and low purchasing power [35].

Achievement of CVD prevention targets among people with diabetes varies by region. This may reflect regional differences in levels of economic development, cultural practices, health care systems, and health policies. For example, regions with higher levels of economic development may have better medical resources and health education. Some regions may have stricter and more effective smoke-free policies, restrictions on alcohol consumption, and promotion of healthy diets. The specific causes of regional differences should be further investigated in future studies.

Achievement of the targets for the different subgroups was also unsatisfactory. In line with previous studies [24,25], older age groups were associated with better achievement of treatment targets but also with poorer achievement of metabolic targets. This may be due to an increased awareness of health risks. Women had poorer achievement of BMI, total cholesterol and physical activity targets but a high prevalence of non-smoking and non-alcohol consumption. Differences in social roles and expectations may contribute to gender differences. People with higher levels of education were more likely to achieve treatment targets but had poorer control of BMI. Overall, these differences may be due to a combination of factors. These included individual behaviour, physical characteristics, socio-cultural background and economic status. Understanding these differences may help to develop more targeted health interventions to improve patients’ overall success with treatment, metabolic, and lifestyle targets. For example, tailored public health policies that address gender- and age-specific challenges can create a supportive environment for improving existing disparities in target attainment.

To improve our interventions to prevent CVD in people with diabetes in LMICs, we recommend the following actions: Establish multidisciplinary teams for care management and decision support to prevent multiple adverse diabetes outcomes [14,36]. Encourage wearable devices that provide real-time feedback to promote proactive health awareness and adherence to CVD risk management guidelines among people with diabetes [37]. Utilise non-physician health professionals to deliver personalised interventions, a cost-effective and scalable management strategy [38]. Implement policies that increase taxes on sugary drinks, reduce the marketing of tobacco, alcohol, and unhealthy foods, and promote a healthy business environment to support zero-level prevention initiatives [39]. In addition, improving the primary health care system, strengthening health education and awareness, implementing community-based interventions and ensuring affordable and accessible medicines are all effective policy recommendations that address the needs and capacities of LMICs.

Our study has several limitations. First, selection bias may have been present in this study because participants who responded positively to recruitment were likely to be predominantly individuals with better health status and disease control. Second, unharmonised data leads to unavoidable heterogeneity, which can result from differences in survey instruments, the size of the population surveyed, and the year in which the survey was conducted. Third, patients’ self-reported history of diabetes and medication history, among others, may be subject to recall bias and social desirability bias, resulting in potential misclassification or overestimation of outcomes. In addition, the survey was conducted in 38 LMICs between 2013–20, which does not reflect the current situation, and the results cannot be extrapolated to other countries and regions. Because of the cross-sectional study design, we could only observe the performance of these countries during the survey year. We could not track changes in target achievement or establish causality. In the future, more LMICs and regions need to be included, and repeated cross-sectional designs or prospective cohort studies need to be conducted wherever possible to explore long-term trends in CVD prevention in people with diabetes in more detail.

## CONCLUSIONS

The achievement of individualised guideline-recommended targets for CVD prevention in people with diabetes in LMICs is inadequate. In line with the goals of the Global Diabetes Compact, our study showed that there is an urgent need to control behavioural and metabolic risk factors in people with diabetes and to ensure access to essential medicines to reduce the risk of CVD and the burden on health systems.

## Additional material


Online Supplementary Document
