# Supplementary material for: Achievement of recommended targets for cardiovascular disease prevention in adults with diabetes in 38 low- and middle-income countries
Source: J Glob Health. 2024 Sep 20;14:04148. doi: 10.7189/jogh.14.04148 (PMC11413614; doi:10.7189/jogh.14.04148)
Supplement: Online Supplementary Document [file jogh-14-04148-s001.pdf]

Online Supplementary Document

Appendix 1: Supplemental methods **2**

Appendix 2: Survey and subpopulation characteristics **5**

Supplementary Table S1: Survey characteristics 5

Supplementary Table S2: Supplementary Table S2: Characteristics of study subpopulation with diabetes 6

Supplementary Table S3: Individual characteristics of the diabetes, high risk of cardiovascular disease (CVD) and established CVD group 7

Appendix 3: Prevalence of treatment, metabolic and lifestyle targets for CVD prevention in patients with diabetes, by region and country 8

Supplementary Table S4: Prevalence of treatment, metabolic and lifestyle targets for CVD prevention in patients with diabetes, by income group 8

Supplementary Table S5: Prevalence of treatment, metabolic and lifestyle targets for CVD prevention in patients with diabetes, by region 10

Supplementary Table S6: Prevalence of treatment, metabolic and lifestyle targets for CVD prevention in patients with diabetes, by country 11

Appendix 4: Prevalence of guideline-recommended targets for all treatment, metabolic and lifestyle components of CVD prevention in patients with diabetes by World Bank income group and region **16**

Supplementary Table S7: Prevalence of guideline-recommended targets for all treatment, metabolic and lifestyle components of CVD prevention in patients with diabetes by World Bank income group and region 16

Appendix 5: Association between individual characteristics and guideline-recommended treatment, metabolic and lifestyle targets in people with diabetes **17**

Supplementary Table S8: Association between individual characteristics and guideline-recommended treatment, metabolic and lifestyle targets in people with diabetes (Univariate Poisson regression model) 17

Supplementary Table S9: Association between individual characteristics and guideline-recommended treatment, metabolic and lifestyle targets in people with diabetes (Multivariable Poisson regression model) 24

Appendix 6: Sensitivity analysis **30**

Supplementary Table S10-S12: Prevalence of treatment, metabolic and lifestyle targets to prevent CVD among people with diabetes using re-adjusted weights for the proportion of the population aged 25-69 in each country 30

Supplementary Table S13-S14: Prevalence of aspirin use in WHO ISH risk >30% subgroup, by income group and region 33

Supplementary Table S15: Prevalence of blood pressure <130/80 mm Hg, by income group and region 35

Appendix 7: STROBE Checklist **36**

Appendix 1: Supplemental methods

The following content complements the methodology section in the main paper.

Inclusion criteria for surveys

1. Since the STEP-wise approach to noncommunicable disease risk factor surveillance (STEPS) after 2013 introduced the question of cardiovascular disease (CVD) history, we selected the survey conducted after 2013.
2. The surveys and studies are all obtained from the STEPS database on the World Health Organization (WHO) official website and have nationally representative individual-level data.
3. Surveys were exclusively carried out in countries classified as upper-middle (UMIC), lower-middle (L-MIC), or low-income (LIC) according to the World Bank's classification for the survey year.
4. For some countries, such as Pakistan and Tonga, they were excluded from our analysis because metabolic biochemical indicators were not collected.
5. Given the specificity of pregnant women, only non-pregnant adults were included in our analysis.

Country classification and characteristics

We grouped countries according to geographical regions defined by the WHO and income groups defined by the World Bank in the year the survey was conducted.

References: World Bank. World Bank Country and Lending Groups – World Bank Data Help Desk. 2019. https://datahelpdesk.worldbank.org/knowledgebase/articles/906519-world-bank-country-and-lending-(accessed May 23, 2024).

Survey introduction

For detailed sampling methods, survey procedures, WHO STEPS questionnaire and introductions for each country, please refer to the following websites (accessed January 3, 2023):

https://extranet.who.int/ncdsmicrodata/index.php/catalog/steps/?page=1&ps=15&repo=STEPS

Data sharing

Data from the 38 countries surveyed in this study are shared and publicly available. After passing the application process, survey files, codebooks and de-identified microdata can be downloaded via the link above or WHO website.

Text of relevant questions in the underlying surveys

1. History of diabetes mellitus

   "Have you ever been told by a doctor or other health worker that you have raised blood sugar or diabetes?"

2. Treatment targets

Hypoglycemic agents: “In the past two weeks, have you taken any drugs (medication) for diabetes prescribed by a doctor or other health worker?”

Statins: “Are you currently taking statins (Lovastatin/Simvastatin/Atorvastatin or any other statin) regularly to prevent or treat heart disease?”

Antihypertensive drugs: “In the past two weeks, have you taken any drugs (medication) for raised blood pressure prescribed by a doctor or other health worker?”

Aspirin: “Are you currently taking aspirin regularly to prevent or treat heart disease?”

3. Lifestyle targets

Tobacco use: “Do you currently smoke any tobacco products, such as cigarettes, cigars or pipes?”

Alcohol consumption: “Have you consumed any alcohol within the past 30 days?”

Dietary fruit and vegetable: “In a typical week, on how many days do you eat fruit?” & “How many servings of fruit do you eat on one of those days?” & “In a typical week, on how many days do you eat vegetables?” & “How many servings of vegetables do you eat on one of those days?”

Physical activity: “Does your work involve vigorous-intensity activity that causes large increases in breathing or heart rate like [carrying or lifting heavy loads, digging or construction work] for at least 10 minutes continuously?” & “In a typical week, on how many days do you do vigorous-intensity activities as part of your work?” & “How much time do you spend doing vigorous-intensity activities at work on a typical day?” & “Does your work involve moderate-intensity activity, that causes small increases in breathing or heart rate such as brisk walking [or carrying light loads] for at least 10 minutes continuously?” & “In a typical week, on how many days do you do moderate-intensity activities as part of your work?” & “How much time do you spend doing moderate-intensity activities at work on a typical day?”. Physical activities included work and recreational activities. The content of the Recreational activity questionnaire is the same as that of work.

Statistical analysis

Continuous variables are presented using means and standard deviations, while categorical variables are conveyed through counts and proportions. For each country in the STEPS dataset, the proportions of CVD secondary prevention behaviors, along with 95% confidence intervals, were computed using sampling weights, stratifications, and primary sampling units provided by the 38 countries. To refine the analysis, new adjusted weights were calculated for each country by utilizing the original sampling weights in the dataset (e.g., Step 1 weights from STEP Survey 1). These adjusted weights were calculated by dividing the sum of the pooled sample weights by the sum of the original weights for each country, and then multiplying the result by the original weight for each individual. The 10-year CVD risk was estimated for each participant according to the WHO CVD risk laboratory-based charts. The WHO CVD risk laboratory-based charts including sex, age, current smoking, systolic blood pressure, diabetes mellitus, and total cholesterol. High-risk population was classified based on a 10-year CVD risk score greater than 20%.

In order to explore individualized characteristics influencing guideline compliance, we computed overall proportions and subgroups (e.g., gender, age groups: 18-34 years, 35-44 years, 45-54 years, 55-69 years. Prevalence of CVD prevention recommendations by World Bank income category, WHO region, and education group). Due to the relatively low prevalence of our indicator of interest among CVD patients within each country, Zou's modified Poisson regression with robust error variance was employed to fit the

multivariable regression model. We report regressions as the absolute difference between the risk ratio and the predicted probability and the average marginal effect. All descriptive statistics and regression models are estimated using a robust error structure with standard errors adjusted for clustering in the primary sample unit. Standard errors were computed using a linear variance estimator via Stata's command set. The treatment variables, metabolic variables, lifestyle variables, and covariates (such as gender and education) that were primarily analyzed had missing values of less than 0.5%. Consequently, we consider these missing data negligible, and they were not separately addressed in the statistical analyses.

The weights were re-adjured in sensitivity analyses. These adjusted weights were derived by dividing the sum of the original weights by the 2019 population aged 25-69, and then multiplying the result by the original weights for each country. In sensitivity analyses, the 10-year CVD risk was estimated for each participant according to the 2007 WHO/ International Society of Hypertension (WHO ISH) CVD risk charts. The WHO ISH CVD risk charts including sex, age, current smoking, systolic blood pressure, diabetes mellitus, and total cholesterol. High-risk population was classified based on a 10-year CVD risk score greater than 30%.

We plotted Figures 1 to 3 using the ggplot2 package in R version 4.2.2. Other analyzes were performed in Stata version 18.0 software, mainly using the “svyset” command.

Appendix 2: Survey and subpopulation characteristics

Supplementary Table S1: Survey characteristics

Country	ISO code	Income group*	Year†	Response rate‡	Sample size§	Proportion of female participants (%)	Median age, years	With establishe d diabetes	Proportion of individuals by country with a history of diabetes¶
**Africa**									
Algeria	DZA	UMIC	2016	94	2308	54.9	42 (34-52)	211	2.63 (2.05, 3.37)
Benin	BEN	LIC	2015	99	3950	52.1	39 (31-49)	40	2.63 (1.75, 3.93)
Botswana	BWA	UMIC	2014	64	2252	69.4	39 (31-50)	76	2.63 (1.76, 3.93)
Cabo Verde	CPV	L-MIC	2020	64	1941	61.5	44 (34-55)	122	2.63 (2.04, 3.38)
Eswatini	SWZ	L-MIC	2014	76	1738	66.8	42 (32-54)	77	2.63 (1.90, 3.64)
Ethiopia	ETH	LIC	2015	96	6376	57.0	37 (30-48)	95	2.63 (1.87, 3.70)
Kenya	KEN	LIC	2015	92	3239	59.2	39 (31-50)	72	2.63 (1.80, 3.84)
Malawi	MWI	LIC	2017	NR	2926	64.4	40 (32-52)	31	2.63 (1.40, 4.90)

Sao Tome and Principe	STP	L-MIC	2019	91	1425	60.4	39 (32-49)	81	0.47 (1.86, 3.72)
Uganda	UGA	LIC	2014	99	2556	58.8	38 (30-48)	36	2.63 (1.72, 4.00)
Zambia	ZMB	L-MIC	2017	74	2392	60.6	40 (32-50)	48	2.63 (1.81, 3.80)
**Americas**									
Ecuador	ECU	UMIC	2018	69	3310	58.6	44 (34-55)	272	2.63 (2.20, 3.14)
Guyana	GUY	L-MIC	2016	77	684	62.4	45 (35-55)	95	2.63 (1.99, 3.47)
**Eastern Mediterranean**									
Afghanistan	AFG	LIC	2018	78	2524	44.7	42 (33-53)	226	2.63 (1.83, 3.76)
Iraq	IRQ	UMIC	2015	99	2836	60.7	42 (34-52)	377	2.63 (2.22, 3.12)
Jordan	JOR	UMIC	2019	95	2741	64.5	43 (35-54)	451	2.63 (2.26, 3.06)
Lebanon	LBN	UMIC	2017	66-74	958	63.2	50 (41-56)	106	2.63 (1.91, 3.61)
Morocco	MAR	L-MIC	2017	89	3692	65.8	45 (36-55)	370	2.63 (2.31, 2.99)
Sudan	SDN	L-MIC	2016	95	5228	63.0	40 (32-50)	323	2.63 (2.09, 3.30)
**Europe**									
Armenia	ARM	L-MIC	2016	42	1368	73.0	49 (36-58)	96	2.63 (1.96, 3.52)
Azerbaijan	AZE	UMIC	2017	97	2259	58.9	49 (37-57)	185	2.63 (2.11, 3.27)
Belarus	BLR	UMIC	2016	87	4391	58.4	49 (39-58)	317	2.63 (2.20, 3.14)
Georgia	GEO	L-MIC	2016	76	2868	72.6	52 (41-61)	234	2.63 (2.17, 3.18)
Kyrgyzstan	KGZ	LIC	2013	100	2455	63.1	44 (34-53)	109	2.63 (1.90, 3.64)
Moldova	MDA	L-MIC	2013	84	3149	64.0	51 (38-59)	302	2.63 (2.17, 3.19)
Tajikistan	TJK	L-MIC	2016	94	2130	58.2	42 (33-52)	75	2.63 (1.91, 3.61)
Turkmenistan	TKM	UMIC	2018	94	3291	56.8	42 (33-52)	80	2.63 (1.89, 3.66)
**Western Pacific**									
Kiribati	KIR	L-MIC	2015	55	899	56.0	41 (32-51)	80	2.63 (1.44, 4.75)
Mongolia	MNG	L-MIC	2019	98	5280	55.3	43 (34-54)	301	2.63 (2.20, 3.15)

Solomon Islands	SLB	L-MIC	2015	58	1397	54.3	42 (34-51)	59	2.63 (1.83, 3.77)
Tokelau	TK	UMIC	2014	70	403	52.6	43 (32-53)	96	2.63 (0.83, 8.07)
Tuvalu	TUV	UMIV	2015	76	824	54.6	47 (34-56)	89	2.63 (0.92, 7.33)
Vietnam	VNM	L-MIC	2015	97	2721	57.3	45 (37-55)	88	2.63 (1.98, 3.49)
**South-East Asia**									
Bangladesh	BGD	L-MIC	2018	84	6049	52.5	40 (33-50)	435	2.63 (2.28, 3.04)
Myanmar	MMR	LIC	2014	94	7540	65.4	45 (36-54)	479	2.63 (1.65, 4.18)
Nepal	NPL	LIC	2019	86	4321	63.8	42 (33-53)	127	2.63 (1.74, 3.96)
Sir Lanka	LKA	L-MIC	2014	72	3736	61.3	46 (37-56)	511	2.63 (2.28, 3.04)
Timor-Leste	TLS	L-MIC	2014	96	1926	56.4	43 (34-54)	17	2.63 (1.55, 4.43)
**Total**	..	..	..	87 (74-95)	110 083	60.0	43 (34-54)	6789	

World regions are defined by WHO. CVD=cardiovascular disease. ISO=International Organization for Standardization. UMIC=Upper-middle-income country. LIC=low-income country. L-MIC=lower middle-income country. *Income groups are defined by the World Bank fiscal year categories in the year the survey was done. †Year reflects the year(s) of survey data collection. ‡ Response rate is equal to the number of complete interviews divided by the total number of surveys. §The sample includes individuals aged 25-69 years of age. ¶ Proportion of individuals by country with a history of diabetes was calculated by new weight to represent each country equally.

Supplementary Table S2: Characteristics of study subpopulation with diabetes

Characteristic	Low-income countries n=9 countries	Lower middle-income countries n=18 countries	Upper-middle-income countries n=11 countries	Overall n=38 countries
Total No.	537	3992	2260	6789
Age, median (IQR), years	51 (43, 60)	54 (45, 61)	55 (47, 62)	54 (45, 61)
Age group, years				

25-44	27.3 (19.7, 36.4)	17.1 (14.7, 19.9)	15.4 (13.0, 18.1)	18.2 (16.2, 20.4)
45-54	34.6 (27.9, 42.0)	40.0 (37.6, 43.3)	36.9 (33.9, 40.0)	38.5 36.4, 40.6)
55-69	38.1 (30.8, 45.9)	42.5 (39.7, 45.2)	47.7 (44.1, 51.4)	43.3 (41.1, 45.5)
Sex				
Female	52.3 (44.3, 60.2)	44.4 (41.4, 47.4)	43.0 (40.2, 45.8)	54.8 (52.5, 57.0)
Male	47.7 (39.8, 55.7)	55.6 (52.6, 58.6)	57.1 (54.2, 59.8)	45.2 (43.0, 47.5)
Education				
No formal schooling	26.3 (20.4, 33.1)	10.0 (8.3, 12.0)	9.5 (5.9, 9.5)	11.9 (10.4, 13.6)
Primary	40.3 (31.9, 49.3)	34.6 (31.5, 37.9)	43.7 (36.4, 51.5)	38.1 (34.9, 41.5)
Secondary or higher	33.4 (26.7, 40.9)	55.4 (52.2, 58.6)	48.7 (41.8, 55.7)	50.0 (46.9, 53.0)
Hypertension				
Yes	66.8 (58.8, 74.0)	56.5 (53.5, 59.4)	61.0 (57.0, 64.8)	59.4 (57.0, 61.7)
Hypercholesterolemia				
Yes	20.8 (15.5, 27.4)	27.9 (25.5, 30.5)	41.2 (37.8, 44.7)	18.2 (16.5, 20.0)

Sampling weights were adjusted to represent each country equally.

Supplementary Table S3: Individual characteristics of the diabetes, high risk of CVD and established CVD group

	Diabetes (N=6789)		10 years CVD risk >20% (N=1150)		With established CVD and diabetes (N=1292)	
Characteristics	Unweighted, n	Weighted, %	Unweighted, n	Weighted, %	Unweighted, n	Weighted, %
**Age**						
25-39 years	878	18.2	3	0.6	104	12.2
40-54 years	2610	38.5	169	7.7	424	15.1
55-69 years	3301	43.3	1401	36.1	764	20.7
**Sex**						

Male	2479	45.2	694	23.7	473	16.7
Female	4310	54.8	879	14.6	819	17.3
**Country economic status**						
Upper-middle	2260	28.9	729	26.6	422	18.6
Lower-middle	3992	55.3	741	16.2	755	15.7
Low	537	15.8	103	13.0	115	18.4
**Region**						
Africa	889	28.9	104	7.6	109	11.4
Americas	367	5.3	3	1.1	48	13.6
Western Pacific	713	15.8	115	10.7	160	16.8
European	1398	21.1	693	44.5	432	29.0
Eastern Mediterranean	1853	15.8	589	30.2	273	15.9
South-East Asia	1569	13.2	69	4.6	270	12.9
**Education**						
No schooling	951	11.9	240	18.8	149	15.3
Primary	2493	38.1	455	13.2	433	15.4
Secondary or higher	3128	50.0	815	22.7	667	18.0
**Area of residence**						
Urban	2599	56.2	683	21.0	533	19.4
Rural	1607	43.8	428	18.3	279	16.2

CVD=cardiovascular. Prevalence was calculated based on the adjusted weight, giving equal weight to each country.

Appendix 3: Prevalence of treatment, metabolic and lifestyle targets for CVD prevention in patients with diabetes, by region and country

Supplementary Table S4: Prevalence of treatment, metabolic and lifestyle targets for CVD prevention in patients with diabetes, by income group

	Prevalence (95% CI)

	Overall	Upper-Middle Income Countries	Lower-Middle Income Countries	Low-Income Countries
**A. Treatment targets**				
Glucose-lowering drug	57.86 (55.42, 60.25)	67.65 (63.67, 71.39)	52.93 (49.61, 56.22)	56.15 (47.18, 64.73)
Statins	11.14 (9.85, 12.58)	17.90 (15.07, 21.13)	8.16 (6.92, 9.60)	8.64 (4.73, 15.27)
Antihypertensive drug	54.72 (51.84, 57.57)	63.04 (58.29, 67.55)	51.77 (48.02, 55.51)	49.77 (39.40, 60.16)
Aspirin	26.52 (22.86, 30.54)	35.20 (30.04, 40.73)	19.63 (15.84, 24.06)	34.75 (20.96, 51.67)
**B. Metabolic targets**				
Fasting blood glucose <6.1 mmol/L	43.67 (41.17, 46.19)	37.40 (33.51, 41.45)	44.87 (41.78, 48.01)	50.92 (42.91, 58.89)
Body mass index <25 kg/m²	27.25 (24.83, 29.80)	15.02 (12.23, 18.31)	29.78 (27.11, 32.61)	40.78 (32.58, 49.52)
Blood pressure <140/90 mm Hg	48.72 (46.53, 50.92)	48.72 (45.60, 51.85)	49.30 (46.54, 52.07)	46.70 (38.53, 55.06)
Total cholesterol <5.0 mmol/L	59.24 (56.91, 61.53)	54.74 (51.01, 58.43)	60.14 (57.25, 62.96)	64.35 (56.60, 71.43)
**C. Lifestyle targets**				
Not currently smoking	83.82 (81.56, 85.86)	78.39 (73.48, 82.61)	83.88 (81.33, 86.13)	93.59 (89.16, 96.29)
Not currently drinking	79.84 (77.86, 81.68)	82.83 (79.31, 85.86)	78.28 (75.67, 80.69)	79.78 (72.59, 85.46)
Sufficient physical activity	53.68 (51.36, 55.99)	45.37 (41.63, 49.17)	54.82 (51.71, 57.90)	64.93 (57.83, 71.43)
Dietary fruit and vegetables ≥5 servings/day	19.51 (17.69, 21.46)	19.59 (16.46, 23.16)	21.08 (18.74, 23.62)	13.86 (8.89, 20.96)

Prevalence was calculated based on the adjusted weight, giving equal weight to each country. The prevalence of statin use was assessed only in people with diabetes

aged 40 years or older. The prevalence of aspirin use was assessed in people with diabetes with a history of CVD and a 10-year risk of CVD greater than 20%, as calculated using the WHO CVD Risk Laboratory-based Chart. Bangladesh, Myanmar, Nepal, Sir Lanka and Timor-Leste have a body mass index target of less than 23 kg/m^2. Total cholesterol targets were set at less than 5.0 mmol/L (190 mg/dL) for patients without a history of CVD and less than 4.0 mmol/L (152 mg/dL) for patients with a history of CVD.

Supplementary Table S5: Prevalence of treatment, metabolic and lifestyle targets for CVD prevention in patients with diabetes, by region

Treatment targets

	Prevalence (95% CI)				
Region	Glucose-lowering drug	Statins	Antihypertensive drug	Aspirin	All 4 targets met
Africa	54.56 (48.93, 60.07)	8.95 (6.40, 12.38)	49.84 (43.29, 56.40)	21.93 (13.31, 33.95)	8.41 (6.03, 11.60)
Americas	63.23 (55.84, 70.04)	8.77 (5.47, 13.75)	58.71 (50.39, 66.55)	25.00 (12.89, 42.87)	8.63 (4.87, 14.84)
Western Pacific	45.49 (38.10, 53.09)	6.66 (4.62, 9.51)	45.14 (37.05, 53.49)	16.90 (11.90, 23.45)	6.30 (4.38, 8.97)
European	58.07 (54.00, 62.04)	11.32 (8.67, 14.66)	58.97 (54.33, 63.46)	38.13 (33.31, 43.20)	7.47 (5.39, 10.25)
Eastern Mediterranean	67.06 (63.42, 70.50)	20.48 (17.26, 24.12)	64.91 (60.68, 68.92)	37.95 (32.03, 44.26)	18.17 (14.79, 22.12)
South-East Asia	67.59 (60.18, 74.21)	10.13 (7.95, 12.83)	56.31 (47.29, 64.94)	16.49 (10.81, 24.36)	11.59 (7.29, 17.92)

Prevalence was calculated using adjusted weight, giving each country equal weight. The prevalence of statin use was assessed only in people with diabetes aged 40 years or older. The prevalence of aspirin use was assessed in people with diabetes with a history of CVD and a 10-year risk of CVD greater than 20%, as calculated using the WHO CVD Risk Laboratory-based Chart.

Metabolic targets

	Prevalence (95% CI)				
Region	Body mass index <25 kg/m²	Blood pressure <140/90 mm Hg	Total cholesterol <5.0 mmol/L	Fasting blood-glucose <6.1 mmol/L	All 4 targets met
Africa	50.58 (45.26, 55.88)	37.22 (32.04, 42.71)	49.84 (44.59, 55.08)	68.21 (63.22, 72.81)	11.97 (7.96, 17.62)
Americas	44.44 (36.97, 52.17)	25.32 (19.10, 32.75)	67.32 (59.70, 74.13)	47.59 (40.70, 54.57)	5.65 (3.32, 9.46)

Western Pacific	43.12 (35.17, 51.44)	22.41 (16.28, 30.01)	56.11 (50.61, 61.47)	49.86 (44.47, 55.25)	6.73 (4.21, 10.60)
European	38.16 (34.53, 41.94)	14.81 (12.13, 17.96)	31.02 (27.56, 34.70)	49.66 (45.52, 53.81)	2.55 (1.66, 3.91)
Eastern Mediterranean	36.79 (33.14, 40.60)	21.02 (18.04, 24.36)	48.78 (44.66, 52.91)	61.07 (56.89, 65.10)	4.23 (2.94, 6.05)
South-East Asia	45.85 (39.56, 52.27)	39.25 (32.79, 46.11)	58.22 (52.23, 63.98)	68.57 (62.10, 74.39)	15.65 (10.15, 23.35)

Prevalence was calculated using adjusted weight, giving each country equal weight. Bangladesh, Myanmar, Nepal, Sir Lanka and Timor-Leste have a body mass index target of less than 23 kg/m^2. Total cholesterol targets were set at less than 5.0 mmol/L (190 mg/dL) for patients without a history of CVD and less than 4.0 mmol/L (152 mg/dL) for patients with a history of CVD.

Lifestyle targets

	Prevalence (95% CI)				
Region	Not currently smoking	Not currently drinking	Sufficient physical activity	Fruit and vegetables ≥5 servings/day	All 4 targets met
Africa	94.75 (92.21, 96.49)	77.51 (72.74, 81.66)	63.68 (58.83, 68.26)	17.27 (13.51, 21.82)	7.06 (4.58, 10.73)
Americas	89.38 (84.75, 92.73)	71.62 (65.31, 77.18)	47.81 (41.11, 54.59)	4.78 (2.63, 8.56)	1.04 (0.17, 5.93)
Western Pacific	65.76 (58.92, 72.01)	74.44 (68.79, 79.38)	58.26 (50.88, 65.29)	15.71 (11.34, 21.37)	6.17 (3.77, 9.93)
European	86.42 (83.23, 89.09)	69.02 (65.02, 72.75)	48.19 (44.02, 52.39)	34.22 (30.50, 38.14)	12.96 (10.47, 15.94)
Eastern Mediterranean	80.71 (77.29, 83.72)	98.86 (97.62, 99.46)	36.16 (32.63, 39.84)	14.26 (12.11, 16.71)	3.82 (2.86, 5.07)
South-East Asia	78.82 (71.80, 84.48)	89.20 (84.89, 92.38)	58.37 (52.19, 64.29)	17.64 (12.91, 23.61)	5.88 (4.51, 7.64)

Prevalence was calculated using adjusted weight, giving each country equal weight.

Supplementary Table S6: Prevalence of treatment, metabolic and lifestyle targets for CVD prevention in patients with diabetes, by country

Treatment targets

	Prevalence (95% CI)				
Country	Glucose-lowering drug	Statins	Antihypertensive drug	Aspirin	All 4 targets met
Afghanistan	65.63 (50.05, 78.44)	16.27 (7.32, 32.33)	78.40 (63.51, 88.33)	47.04 (24.66, 70.69)	23.41 (13.74, 36.96)

Algeria	79.14 (72.57, 84.47)	24.87 (18.96, 31.89)	53.86 (46.02, 61.52)	37.32 (26.90, 49.07)	18.77 (14.24, 24.34)
Armenia	62.95 (49.32, 74.79)	5.66 (2.02, 14.86)	44.50 (31.81, 57.95)	12.62 (5.58, 26.07)	2.01 (0.57, 6.85)
Azerbaijan	76.28 (67.09, 83.53)	9.66 (5.12, 17.49)	60.10 (51.09, 68.47)	30.15 (19.62, 43.28)	8.58 (4.66, 15.26)
Bangladesh	85.98 (77.85, 91.46)	13.20 (7.82, 21.43)	63.98 (55.06, 72.03)	27.68 (16.38, 42.78)	9.75 (5.91, 15.66)
Belarus	50.13 (43.68, 56.59)	11.09 (7.25, 16.58)	70.86 (64.27, 76.67)	38.85 (32.16, 46.00)	8.39 (5.21, 13.24)
Benin	72.79 (54.58, 85.62)	8.19 (1.22, 39.20)	61.58 (38.00, 80.74)	29.86 (6.12, 73.53)	10.84 (2.76, 34.25)
Botswana	60.05 (47.01, 71.82)	4.91 (1.47, 15.17)	72.97 (59.64, 83.14)	2.81 (0.35, 19.07)	6.94 (2.10, 20.61)
Cabo Verde	72.89 (63.03, 80.92)	26.50 (17.03, 38.78)	75.07 (63.02, 84.18)	44.70 (23.52, 68.00)	22.26 (13.73, 33.99)
Ecuador	50.35 (42.91, 57.78)	9.75 (5.76, 16.05)	57.79 (48.29, 66.75)	15.56 (6.53, 32.72)	6.28 (3.40, 11.30)
Eswatini	68.01 (53.69, 79.59)	4.58 (0.94, 19.60)	57.77 (41.08, 72.86)	22.89 (4.20, 66.75)	3.19 (1.04, 9.38)
Ethiopia	67.85 (51.63, 80.68)	6.28 (1.05, 29.66)	23.53 (12.00, 40.99)	2.75 (0.34, 19.19)	10.50 (3.95, 25.05)
Georgia	62.12 (53.66, 69.90)	12.30 (7.45, 19.63)	70.25 (60.14, 78.71)	22.20 (15.19, 31.24)	6.21 (3.15, 11.86)
Guyana	75.22 (62.91, 84.46)	7.82 (3.44, 16.82)	59.62 (45.85, 72.03)	34.43 (13.64, 63.58)	10.99 (4.77, 23.31)
Iraq	72.76 (66.48, 78.24)	8.25 (5.24, 12.74)	62.03 (55.15, 68.45)	27.65 (19.94, 36.96)	6.61 (4.30, 10.03)
Jordan	83.23 (77.72, 87.60)	36.11 (29.08, 43.80)	74.23 (66.72, 80.53)	63.60 (53.26, 72.83)	29.87 (23.44, 37.19)
Kenya	40.00 (23.49, 59.15)	0.63 (0.09, 4.50)	21.96 (11.53, 37.78)	4.51 (0.56, 28.41)	3.75 (1.29, 10.41)
Kiribati	56.53 (27.89, 81.39)	1.39 (0.31, 6.12)	28.81 (13.53, 51.13)	23.72 (6.45, 58.37)	4.92 (1.84, 12.53)
Kyrgyzstan	52.08 (37.90, 65.93)	6.81 (2.50, 17.21)	52.18 (36.98, 66.99)	46.38 (32.56, 60.78)	5.28 (1.75, 14.86)
Lebanon	89.24 (78.25, 95.03)	47.16 (35.92, 58.70)	82.60 (71.81, 89.84)	58.50 (43.67, 71.94)	38.11 (26.66, 51.05)
Malawi	46.41 (17.55, 77.89)	13.85 (2.80, 47.26)	46.31 (14.91, 80.94)	75.21 (30.61, 95.43)	10.26 (1.74, 42.40)
Moldova	34.34 (28.16, 41.09)	11.16 (6.68, 18.06)	56.90 (48.44, 64.98)	36.92 (29.65, 44.84)	5.07 (2.71, 9.29)
Mongolia	29.30 (23.50, 35.86)	10.36 (6.71, 15.68)	68.04 (59.13, 75.81)	34.55 (25.89, 44.37)	5.62 (3.39, 9.18)
Morocco	19.56 (15.46, 24.42)	6.80 (4.53, 10.08)	47.28 (40.81, 53.83)	9.44 (5.34, 16.15)	4.28 (2.35, 7.66)
Myanmar	68.36 (61.50, 74.51)	5.90 (3.64, 9.43)	49.53 (35.85, 63.28)	12.82 (7.82, 20.31)	5.56 (3.30, 9.23)
Nepal	77.59 (51.11, 91.98)	1.71 (0.58, 4.94)	36.19 (21.80, 53.57)	0.00 (0.00, 0.00)	4.14 (1.52, 10.78)
Sao Tome and Principe	24.27 (14.42, 37.86)	0.00 (0.00, 0.00)	50.60 (37.07, 64.05)	0.00 (0.00, 0.00)	3.24 (0.64, 14.82)

Solomon Islands	7.30 (2.70, 18.29)	0.74 (0.10, 5.26)	17.43 (7.72, 34.74)	2.67 (0.35, 17.77)	2.32 (0.33, 14.65)
Sri Lanka	68.46 (63.44, 73.08)	25.71 (21.34, 30.62)	56.49 (50.47, 62.32)	41.97 (29.65, 55.39)	19.33 (15.65, 23.64)
Sudan	71.65 (65.80, 76.85)	7.81 (4.45, 13.35)	44.94 (36.66, 53.50)	21.50 (12.95, 33.51)	6.74 (4.64, 9.69)
Tajikistan	54.43 (40.29, 67.89)	6.93 (1.90, 22.31)	51.54 (35.40, 67.36)	36.24 (20.70, 55.31)	3.39 (1.06, 10.36)
Timor-Leste	39.08 (15.56, 69.07)	0.00 (0.00, 0.00)	75.38 (39.07, 93.60)	0.00 (0.00, 0.00)	19.15 (4.89, 52.18)
Tokelau	63.23 (54.91, 70.83)	15.10 (9.69, 22.77)	62.73 (59.45, 65.89)	31.04 (21.09, 43.12)	15.02 (8.92, 24.18)
Turkmenistan	74.90 (60.32, 85.42)	26.34 (14.10, 43.79)	65.45 (48.79, 79.01)	81.69 (65.83, 91.18)	20.81 (10.05, 38.19)
Tuvalu	45.13 (34.41, 56.33)	1.22 (0.43, 3.42)	30.85 (18.53, 46.65)	0.00 (0.00, 0.00)	5.31 (2.77, 9.95)
Uganda	31.25 (15.43, 53.12)	2.93 (0.40, 18.56)	36.62 (16.37, 63.04)	7.26 (0.90, 40.27)	2.33 (0.32, 15.08)
Vietnam	72.67 (58.87, 83.16)	11.62 (6.40, 20.18)	62.97 (41.69, 80.17)	9.43 (3.55, 22.76)	4.61 (2.12, 9.70)
Zambia	34.86 (19.88, 53.58)	2.81 (0.81, 9.24)	47.97 (25.94, 70.82)	13.93 (2.73, 48.28)	0.43 (0.06, 3.06)

The estimates account for weighting and survey design. The prevalence of statin use was assessed only in people with diabetes aged 40 years or older. The prevalence of aspirin use was assessed in people with diabetes with a history of CVD and a 10-year risk of CVD greater than 20%, as calculated using the WHO CVD Risk Laboratory-based Chart.

Metabolic targets

	Prevalence (95% CI)				
Country	Body mass index <25 kg/m²	Blood pressure <140/90 mm Hg	Total cholesterol <5.0 mmol/L	Fasting blood-glucose <6.1 mmol/L	All 4 targets met
Afghanistan	47.90 (35.35, 60.72)	21.48 (12.26, 34.88)	38.40 (23.99, 55.18)	59.86 (44.19, 73.75)	6.78 (2.46, 17.36)
Algeria	30.99 (24.32, 38.55)	21.62 (16.60, 27.65)	56.32 (49.71, 62.72)	70.64 (63.33, 77.03)	3.96 (1.72, 8.84)
Armenia	36.38 (24.57, 50.09)	19.32 (10.56, 32.68)	44.00 (31.58, 57.22)	70.76 (58.98, 80.29)	3.34 (1.33, 8.15)
Azerbaijan	22.47 (15.61, 31.23)	13.51 (8.85, 20.08)	38.17 (29.33, 47.86)	38.23 (29.93, 47.27)	2.41 (0.75, 7.52)
Bangladesh	36.32 (28.80, 44.57)	29.67 (23.31, 36.93)	58.44 (51.41, 65.15)	56.90 (48.44, 64.97)	5.96 (3.15, 10.99)
Belarus	53.01 (46.40, 59.51)	12.33 (8.68, 17.22)	27.10 (21.36, 33.71)	48.66 (42.01, 55.35)	3.18 (1.64, 6.10)
Benin	46.27 (28.49, 65.06)	38.24 (22.33, 57.14)	43.07 (25.71, 62.31)	50.16 (32.00, 68.27)	12.48 (3.81, 33.89)

Botswana	54.04 (40.96, 66.59)	24.64 (13.21, 41.26)	44.82 (32.36, 57.96)	86.46 (74.12, 93.43)	2.23 (0.53, 8.81)
Cabo Verde	34.48 (25.16, 45.16)	31.41 (22.47, 41.98)	51.53 (40.94, 61.98)	65.20 (55.22, 74.01)	3.65 (1.66, 7.83)
Ecuador	52.08 (44.54, 59.52)	22.86 (17.12, 29.84)	78.78 (72.33, 84.05)	57.54 (50.15, 64.60)	7.26 (4.13, 12.45)
Eswatini	37.16 (24.31, 52.13)	14.49 (7.58, 25.93)	43.07 (28.53, 58.90)	79.76 (68.85, 87.53)	4.67 (1.63, 12.71)
Ethiopia	36.21 (23.15, 51.68)	56.15 (41.06, 70.17)	55.15 (40.97, 68.55)	81.79 (67.67, 90.60)	18.74 (9.78, 32.92)
Georgia	43.87 (36.09, 51.96)	8.70 (5.10, 14.44)	36.75 (29.70, 44.42)	52.38 (44.54, 60.10)	0.76 (0.11, 5.17)
Guyana	36.80 (24.83, 50.66)	27.78 (17.38, 41.29)	55.87 (43.15, 67.86)	37.65 (27.21, 49.38)	4.04 (1.38, 11.23)
Iraq	33.97 (27.69, 40.86)	12.42 (8.78, 17.29)	42.28 (36.19, 48.62)	43.69 (37.11, 50.51)	0.31 (0.07, 1.44)
Jordan	47.59 (40.80, 54.48)	8.34 (5.31, 12.89)	62.00 (55.16, 68.39)	76.22 (69.64, 81.75)	3.53 (1.81, 6.79)
Kenya	56.42 (36.98, 74.07)	33.18 (20.62, 48.70)	33.61 (20.04, 50.56)	79.28 (65.07, 88.71)	11.37 (4.54, 25.72)
Kiribati	17.89 (7.96, 35.42)	8.87 (4.21, 17.74)	44.33 (33.77, 55.43)	55.67 (40.32, 70.01)	2.11 (0.51, 8.38)
Kyrgyzstan	36.71 (25.69, 49.31)	16.08 (8.84, 27.45)	28.94 (18.48, 42.24)	48.28 (34.73, 62.10)	2.34 (0.51, 9.96)
Lebanon	34.17 (23.00, 47.44)	26.18 (16.93, 38.17)	57.47 (45.60, 68.54)	37.66 (26.29, 50.57)	2.53 (0.85, 7.30)
Malawi	64.07 (37.44, 84.16)	54.41 (26.03, 80.19)	77.20 (46.85, 92.86)	63.86 (35.40, 85.07)	24.14 (4.17, 69.94)
Moldova	52.83 (46.29, 59.28)	14.76 (9.89, 21.46)	26.40 (20.28, 33.58)	41.75 (34.71, 49.15)	3.71 (1.83, 7.37)
Mongolia	40.09 (33.78, 46.75)	22.87 (17.99, 28.61)	65.53 (58.99, 71.54)	53.49 (46.41, 60.44)	8.43 (5.37, 13.00)
Morocco	28.01 (23.27, 33.31)	23.46 (18.87, 28.78)	50.01 (44.44, 55.57)	84.78 (80.57, 88.21)	4.74 (2.49, 8.85)
Myanmar	32.84 (27.69, 38.44)	33.27 (28.69, 38.19)	54.02 (48.38, 59.56)	43.32 (37.53, 49.29)	8.73 (5.03, 14.73)
Nepal	25.94 (14.39, 42.19)	41.43 (27.28, 57.15)	57.94 (45.05, 69.84)	78.42 (64.79, 87.78)	10.29 (2.19, 37.02)
Sao Tome and Principe	58.35 (44.66, 70.86)	34.45 (24.52, 45.96)	45.10 (34.07, 56.63)	9.73 (4.34, 20.41)	0.55 (0.08, 3.93)
Solomon Islands	78.67 (66.49, 87.27)	26.55 (16.18, 40.35)	62.69 (47.27, 75.90)	43.76 (31.00, 57.41)	10.42 (3.78, 25.66)
Sri Lanka	50.12 (44.91, 55.32)	34.30 (29.80, 39.11)	56.07 (51.12, 60.90)	75.76 (70.86, 80.06)	10.33 (7.79, 13.59)
Sudan	29.11 (23.34, 35.65)	34.24 (28.52, 40.46)	42.51 (35.75, 49.55)	64.21 (56.31, 71.40)	7.49 (4.57, 12.03)
Tajikistan	28.15 (17.30, 42.33)	18.93 (9.71, 33.63)	19.64 (11.36, 31.78)	51.66 (36.04, 66.96)	1.08 (0.23, 4.90)
Timor-Leste	84.02 (62.37, 94.35)	57.58 (31.15, 80.29)	64.63 (37.98, 84.50)	88.44 (49.95, 98.32)	42.92 (20.61, 68.52)
Tokelau	13.44 (8.78, 20.02)	2.56 (1.29, 4.99)	60.48 (55.40, 65.34)	37.98 (28.21, 48.83)	0.00 (0.00, 0.00)

Turkmenistan	31.90 (20.77, 45.56)	14.88 (7.80, 26.54)	27.18 (17.19, 40.16)	45.60 (31.54, 60.40)	3.60 (0.82, 14.45)
Tuvalu	37.73 (30.83, 45.16)	5.90 (3.04, 11.14)	41.35 (33.50, 49.67)	59.51 (48.06, 70.01)	0.49 (0.06, 4.03)
Uganda	74.38 (52.59, 88.37)	58.29 (36.88, 76.98)	37.45 (20.82, 57.69)	82.18 (66.57, 91.44)	25.20 (12.00, 45.42)
Vietnam	70.90 (54.88, 83.00)	67.70 (52.33, 80.01)	62.29 (47.85, 74.84)	48.74 (35.34, 62.32)	18.92 (11.31, 29.92)
Zambia	64.00 (46.11, 78.69)	42.53 (26.12, 60.77)	60.89 (41.54, 77.34)	81.24 (64.01, 91.34)	24.67 (11.92, 44.20)

The estimates account for weighting and survey design. Bangladesh, Myanmar, Nepal, Sir Lanka and Timor-Leste have a body mass index target of less than 23 kg/m2. Total cholesterol targets were set at less than 5.0 mmol/L (190 mg/dL) for patients without a history of CVD and less than 4.0 mmol/L (152 mg/dL) for patients with a history of CVD.

Lifestyle targets

	Prevalence (95% CI)				
Country	Not currently smoking	Not currently drinking	Sufficient physical activity	Fruit and vegetables ≥5 servings/day	All 4 targets met
Afghanistan	93.82 (81.32, 98.15)	0.00 (0.00, 0.00)	39.06 (27.63, 51.83)	1.02 (0.19, 5.30)	0.09 (0.01, 0.68)
Algeria	91.30 (84.79, 95.18)	99.50 (96.59, 99.93)	37.44 (30.46, 44.98)	18.95 (12.50, 27.66)	7.80 (4.27, 13.83)
Armenia	72.44 (56.23, 84.32)	58.63 (44.64, 71.35)	44.23 (31.84, 57.37)	17.10 (9.79, 28.15)	7.57 (3.10, 17.34)
Azerbaijan	90.25 (84.74, 93.92)	90.85 (84.88, 94.61)	41.29 (33.07, 50.04)	36.53 (27.99, 46.02)	12.12 (7.81, 18.33)
Bangladesh	82.98 (75.07, 88.75)	99.96 (99.70, 99.99)	60.41 (52.28, 68.01)	14.10 (9.44, 20.54)	6.86 (4.07, 11.34)
Belarus	83.09 (77.45, 87.55)	51.52 (44.85, 58.14)	44.58 (37.44, 51.96)	26.64 (21.12, 33.00)	6.20 (3.85, 9.84)
Benin	93.56 (66.16, 99.08)	80.26 (58.14, 92.25)	59.94 (41.30, 76.08)	6.57 (1.74, 21.85)	2.64 (0.51, 12.49)
Botswana	96.44 (88.80, 98.93)	84.37 (70.56, 92.40)	58.76 (46.49, 70.04)	11.71 (4.32, 28.03)	6.00 (1.21, 24.99)
Cabo Verde	92.28 (84.33, 96.37)	68.13 (57.53, 77.13)	44.19 (33.39, 55.56)	20.09 (12.97, 29.77)	7.67 (3.99, 14.25)
Ecuador	84.61 (77.90, 89.55)	63.17 (55.93, 69.85)	60.31 (53.46, 66.79)	6.39 (3.44, 11.54)	0.19 (0.03, 1.38)
Eswatini	98.00 (90.74, 99.59)	93.25 (84.52, 97.21)	63.29 (46.94, 77.07)	10.20 (3.31, 27.35)	3.02 (1.06, 8.35)
Ethiopia	96.19 (85.50, 99.09)	72.32 (56.06, 84.26)	66.84 (54.27, 77.39)	10.23 (3.66, 25.50)	9.09 (2.92, 24.95)
Georgia	76.03 (67.42, 82.93)	70.04 (62.01, 77.00)	47.51 (39.04, 56.12)	41.10 (33.14, 49.56)	15.38 (10.53, 21.92)

Guyana	94.16 (86.48, 97.60)	80.07 (69.60, 87.58)	35.31 (25.66, 46.32)	3.18 (0.87, 11.01)	1.88 (0.26, 12.19)
Iraq	76.82 (70.24, 82.32)	99.96 (99.68, 99.99)	16.12 (12.36, 20.76)	18.12 (13.62, 23.70)	2.57 (1.33, 4.92)
Jordan	68.56 (61.49, 74.85)	99.18 (96.83, 99.79)	46.45 (39.74, 53.29)	19.32 (14.45, 25.35)	8.04 (4.82, 13.11)
Kenya	86.24 (70.31, 94.31)	85.01 (67.29, 93.99)	73.25 (53.74, 86.58)	12.62 (5.11, 27.91)	9.81 (3.23, 26.16)
Kiribati	60.48 (49.99, 70.08)	90.49 (70.71, 97.40)	31.30 (14.44, 55.15)	0.92 (0.13, 6.50)	0.00 (0.00, 0.00)
Kyrgyzstan	83.96 (71.49, 91.61)	53.17 (38.99, 66.85)	58.70 (45.03, 71.16)	25.22 (15.82, 37.69)	5.06 (2.37, 10.49)
Lebanon	56.83 (45.38, 67.60)	94.35 (87.40, 97.57)	14.83 (7.87, 26.19)	16.88 (10.17, 26.70)	0.82 (0.11, 5.63)
Malawi	95.73 (73.90, 99.44)	91.18 (56.50, 98.80)	92.76 (71.07, 98.52)	11.25 (2.25, 41.07)	2.14 (0.48, 9.08)
Moldova	91.48 (86.20, 94.86)	40.89 (33.79, 48.40)	56.84 (48.74, 64.60)	33.29 (26.16, 41.27)	8.88 (5.25, 14.63)
Mongolia	70.17 (63.02, 76.45)	56.48 (49.68, 63.05)	56.40 (49.73, 62.86)	13.49 (9.55, 18.71)	4.99 (2.72, 8.96)
Morocco	94.55 (91.24, 96.66)	99.67 (97.72, 99.95)	46.57 (41.08, 52.15)	20.25 (16.27, 24.92)	7.53 (5.19, 10.80)
Myanmar	86.00 (81.85, 89.32)	87.22 (78.88, 92.58)	50.79 (43.32, 58.21)	19.72 (9.56, 36.34)	6.53 (3.38, 12.25)
Nepal	76.65 (63.69, 86.00)	91.29 (83.55, 95.58)	62.80 (48.04, 75.51)	7.88 (2.20, 24.51)	2.72 (0.76, 9.25)
Sao Tome and Principe	98.06 (92.48, 99.52)	32.08 (22.13, 43.96)	69.14 (57.68, 78.65)	49.27 (34.37, 64.30)	7.42 (3.39, 15.48)
Solomon Islands	75.92 (62.27, 85.76)	94.90 (87.41, 98.04)	81.25 (66.76, 90.33)	17.01 (9.48, 28.64)	12.25 (5.46, 25.25)
Sri Lanka	86.49 (82.01, 89.99)	78.80 (73.84, 83.04)	58.60 (53.29, 63.72)	32.37 (27.04, 38.19)	13.28 (10.44, 16.76)
Sudan	93.65 (89.15, 96.36)	0.00 (0.00, 0.00)	53.90 (46.33, 61.30)	9.96 (6.36, 15.28)	3.85 (1.72, 8.39)
Tajikistan	95.73 (84.35, 98.94)	99.77 (98.36, 99.97)	33.83 (21.76, 48.46)	44.55 (30.87, 59.10)	21.98 (11.61, 37.68)
Timor-Leste	62.01 (36.04, 82.54)	88.70 (63.45, 97.26)	59.23 (34.77, 79.84)	14.11 (3.16, 45.24)	0.00 (0.00, 0.00)
Tokelau	40.40 (27.95, 54.22)	58.43 (45.62, 70.20)	68.63 (62.82, 73.92)	5.58 (3.62, 8.51)	1.03 (0.20, 5.08)
Turkmenistan	98.38 (93.06, 99.64)	87.26 (71.83, 94.84)	58.55 (43.31, 72.30)	49.31 (34.73, 64.01)	26.49 (16.85, 39.04)
Tuvalu	75.64 (67.29, 82.41)	82.56 (75.93, 87.66)	52.13 (46.37, 57.84)	6.11 (3.62, 10.14)	0.00 (0.00, 0.00)
Uganda	98.28 (92.80, 99.61)	81.76 (63.17, 92.13)	72.31 (51.80, 86.38)	28.84 (11.90, 54.87)	20.95 (6.30, 51.10)
Vietnam	71.98 (56.10, 83.77)	63.79 (49.29, 76.14)	59.86 (45.15, 72.98)	51.16 (37.35, 64.80)	18.74 (11.32, 29.41)
Zambia	96.15 (84.82, 99.11)	64.76 (44.14, 81.03)	62.56 (43.66, 78.27)	10.25 (4.32, 22.38)	1.13 (0.15, 7.77)

The estimates account for weighting and survey design.

Appendix 4: Prevalence of guideline-recommended targets for all treatment, metabolic and lifestyle components of CVD prevention in patients with diabetes by World Bank income group and region

Supplementary Table S7: Prevalence of guideline-recommended targets for all treatment, metabolic and lifestyle components of CVD prevention in patients with diabetes by World Bank income group and region

By World Bank income group

	Prevalence (95% CI)			
	Overall	Upper-Middle Income Countries	Lower-Middle Income Countries	Low-Income Countries
Treatment targets all met	9.85 (8.54, 11.33)	14.97 (12.52, 17.81)	7.00 (5.57, 8.76)	10.43 (6.45, 16.46)
Metabolic targets all met	8.09 (6.47, 10.07)	2.68 (1.86, 3.86)	8.96 (7.08, 11.28)	14.95 (8.61, 24.68)
Lifestyle targets all met	7.18 (6.06, 8.48)	6.48 (4.83, 8.63)	7.69 (6.45, 9.15)	6.66 (3.20, 13.35)

Prevalence has been recalculated based on the adjusted weight. This gives equal weight to each country.

By region

	Prevalence (95% CI)					
	Africa	Americas	Western Pacific	Europe	Eastern Mediterranean	South-East Asia
Treatment targets all met	8.41 (6.03, 11.60)	8.63 (4.87, 14.84)	6.30 (4.38, 8.97)	7.47 (5.39, 10.25)	18.17 (14.79, 22.12)	11.59 (7.29, 17.92)
Metabolic targets all met	11.97 (7.96, 17.62)	5.65 (3.32, 9.46)	6.73 (4.21, 10.60)	2.55 (1.66, 3.91)	4.23 (2.94, 6.05)	15.65 (10.15, 23.35)
Lifestyle targets all met	7.06 (4.58, 10.73)	1.04 (0.17, 5.93)	6.17 (3.77, 9.93)	12.96 (10.47, 15.94)	3.82 (2.86, 5.07)	5.88 (4.51, 7.64)

Prevalence has been recalculated based on the adjusted weight. This gives equal weight to each country.

Appendix 5: Association between individual characteristics and guideline-recommended treatment, metabolic and lifestyle targets in people with diabetes

Supplementary Table S8: Association between individual characteristics and guideline-recommended treatment, metabolic and lifestyle targets in people with diabetes

(Univariate Poisson regression model)

Treatment targets

	Prevalence (%)	Estimated average marginal effect (95% CI)	*P* values	Risk ratio (95% CI)	*P* values
**Glucose-lowering drug**					
**Age, years**					
25-44	37.41 (30.78, 44.56)	0 (ref)		1 (ref)	
45-54	57.95 (54.29, 61.51)	18.70 (11.52, 25.87)	<0.0001	1.48 (1.24, 1.75)	<0.0001
55-69	65.46 (62.23, 68.56)	24.91 (17.90, 31.92)	<0.0001	1.63 (1.38, 1.93)	<0.0001
**Sex**					
Male	58.98 (55.02, 62.83)	0 (ref)		1 (ref)	
Female	56.94 (53.90, 59.93)	-0.28 (-4.61, 4.04)	0.898	1.00 (0.92, 1.07)	0.898
**Education**					
No schooling	51.70 (44.72, 58.61)	0 (ref)		1 (ref)	
Primary	60.21 (55.79, 64.46)	9.59 (1.73, 17.45)	0.017	1.19 (1.02, 1.37)	0.023
Secondary or higher	57.16 (53.90, 60.36)	4.49 (-3.39, 12.37)	0.264	1.09 (0.94, 1.26)	0.275
**Statins**					
**Age, years**					
40-54	8.10 (6.51, 10.04)	0 (ref)		1 (ref)	
55-70	13.85 (12.02, 15.92)	4.64 (2.33, 6.94)	<0.0001	1.54 (1.23, 1.93)	<0.0001
**Sex**					
Male	11.37 (9.45, 13.62)	0 (ref)		1 (ref)	
Female	10.96 (9.40, 12.75)	0.01 (-2.23, 2.25)	0.992	1.00 (0.82, 1.22)	0.992
**Education**					
No schooling	10.22 (6.73, 15.24)	0 (ref)		1 (ref)	

Primary	10.07 (8.24, 12.26)	0.22 (-4.98, 5.42)	0.934	1.02 (0.61, 1.73)	0.934
Secondary or higher	11.84 (10.01, 13.95)	2.06 (-2.52, 6.64)	0.378	1.21 (0.77, 1.90)	0.412
**Antihypertensive drug**					
**Age, years**					
25-44	16.11 (11.78, 21.65)	0 (ref)		1 (ref)	
45-54	34.49 (31.46, 37.66)	21.60 (12.42, 30.77)	<0.0001	1.70 (1.28, 2.27)	<0.0001
55-70	52.83 (49.66, 55.98)	32.58 (23.08, 42.08)	<0.0001	2.06 (1.54, 2.77)	<0.0001
**Sex**					
Male	33.51 (30.32, 36.86)	0 (ref)		1 (ref)	
Female	43.68 (40.92, 46.48)	11.29 (6.40, 16.18)	<0.0001	1.23 (1.12, 1.36)	<0.0001
**Education**					
No schooling	38.24 (32.53, 44.30)	0 (ref)		1 (ref)	
Primary	37.29 (33.59, 41.14)	6.90 (-2.83, 16.62)	0.164	1.14 (0.94, 1.37)	0.180
Secondary or higher	40.49 (37.54, 43.51)	3.29 (-7.01, 13.59)	0.531	1.07 (0.87, 1.30)	0.538
**Aspirin**					
**Age, years**					
25-44	9.34 (3.72, 21.55)	0 (ref)		1 (ref)	
45-54	25.94 (20.26, 32.56)	13.01 (3.61, 22.40)	0.007	2.04 (1.03, 4.04)	0.041
55-70	29.18 (24.42, 34.44)	15.80 (6.80, 24.81)	0.001	2.26 (1.15, 4.45)	0.018
**Sex**					
Male	23.52 (18.91, 28.85)	0 (ref)		1 (ref)	
Female	29.55 (24.21, 35.53)	3.88 (-1.72, 9.48)	0.174	1.16 (0.93, 1.44)	0.180
**Education**					
No schooling	29.28 (16.05, 47.29)	0 (ref)		1 (ref)	
Primary	21.79 (16.75, 27.84)	8.53 (-0.87, 17.93)	0.075	1.41 (0.95, 2.11)	0.089
Secondary or higher	31.13 (26.77, 35.87)	7.38 (-0.76, 15.52)	0.075	1.36 (0.94, 1.97)	0.105

Prevalence has been recalculated based on the adjusted weight. This gives equal weight to each country. The prevalence of statin use was assessed only in people with diabetes aged 40 years or older. The prevalence of aspirin use was assessed in people with diabetes with a history of CVD and a 10-year risk of CVD greater than 20%, as calculated using the WHO CVD Risk Laboratory-based Chart.

Metabolic targets

	Prevalence (%)	Estimated average marginal effect (95% CI)	*P* values	Risk ratio (95% CI)	*P* values
**Fasting blood-glucose <6.1 mmol/L**					
**Age, years**					
25-44	63.10 (56.70, 69.07)	0 (ref)		1 (ref)	
45-54	39.39 (36.06, 42.82)	-19.38 (-25.85, -12.91)	<0.0001	0.68 (0.60, 0.76)	<0.0001
55-69	39.28 (36.01, 42.65)	-20.33 (-26.87, -13.79)	<0.0001	0.66 (0.58, 0.75)	<0.0001
**Sex**					
Male	43.68 (39.91, 47.52)	0 (ref)		1 (ref)	
Female	43.65 (40.51, 46.86)	-0.24 (-4.37, 3.90)	0.911	0.90 (1.09, 0.91)	0.898
**Education**					
No schooling	47.05 (40.71, 53.49)	0 (ref)		1 (ref)	
Primary	42.55 (37.73, 47.51)	-5.53 (-12.57, 1.51)	0.123	0.88 (0.75, 1.03)	0.115
Secondary or higher	43.35 (40.46, 46.29)	-1.66 (-8.48, 5.17)	0.634	0.96 (0.83, 1.12)	0.631
**Body mass index < 25 kg/m²**					
**Age, years**					
25-44	40.69 (33.58, 48.21)	0 (ref)		1 (ref)	
45-54	23.68 (20.77, 26.86)	-11.78 (-18.14, -5.42)	<0.0001	0.67 (0.55, 0.82)	<0.0001
55-70	24.76 (21.79, 27.98)	-10.38 (-16.73, -4.02)	0.001	0.71 (0.59, 0.86)	0.001
**Sex**					
Male	34.03 (30.16, 38.12)	0 (ref)		1 (ref)	

Female	21.64 (19.20, 24.30)	-11.53 (-15.43, -7.63)	<0.0001	0.66 (0.57, 0.75)	<0.0001
**Education**					
No schooling	35.15 (29.03, 41.80)	0 (ref)		1 (ref)	
Primary	28.09 (23.39, 33.32)	-6.29 (-13.51, 0.92)	0.087	0.81 (0.64, 1.02)	0.074
Secondary or higher	24.36 (21.78, 27.14)	-7.86 (-15.13, -0.59)	0.034	0.76 (0.60, 0.96)	0.024
**Blood pressure < 140/90 mm Hg**					
**Age, years**					
25-44	68.35 (62.35, 73.80)	0 (ref)		1 (ref)	
45-54	50.97 (47.57, 54.37)	-13.83 (-20.07, -7.59)	<0.0001	0.79 (0.71, 0.87)	<0.0001
55-70	38.46 (35.51, 41.49)	-26.43 (-32.34, -20.51)	<0.0001	0.60 (0.53, 0.66)	<0.0001
**Sex**					
Male	49.79 (46.17, 53.41)	0 (ref)		1 (ref)	
Female	47.84 (45.27, 50.42)	-1.38 (-5.33, 2.58)	0.495	0.97 (0.90, 1.05)	0.493
**Education**					
No schooling	48.32 (41.89, 54.81)	0 (ref)		1 (ref)	
Primary	52.49 (48.53, 56.42)	2.19 (-5.31, 9.69)	0.567	1.05 (0.89, 1.23)	0.572
Secondary or higher	46.00 (43.10, 48.93)	3.25 (-4.38, 10.89)	0.404	1.07 (0.91, 1.26)	0.412
**Total cholesterol <5.0 mmol/L**					
**Age, years**					
25-44	63.97 (57.60, 69.89)	0 (ref)		1 (ref)	
45-54	61.88 (58.42, 65.21)	0.29 (-5.36, 5.94)	0.919	1.00 (0.92, 1.10)	0.919
55-70	54.91 (51.79, 57.99)	-6.85 (-12.64, -1.05)	0.021	0.89 (0.81, 0.98)	0.018
**Sex**					
Male	66.50 (63.11, 69.73)	0 (ref)		1 (ref)	
Female	53.24 (50.42, 56.04)	-11.03 (-14.72, -7.33)	<0.0001	0.83 (0.78, 0.88)	<0.0001
**Education**					

No schooling	63.29 (56.60, 69.50)	0 (ref)		1 (ref)	
Primary	58.91 (54.58, 63.11)	1.39 (-5.73, 8.52)	0.701	1.02 (0.91, 1.16)	0.703
Secondary or higher	58.73 (55.78, 61.61)	1.44 (-5.95, 8.83)	0.702	1.02 (0.90, 1.16)	0.704

Prevalence has been recalculated based on the adjusted weight. This gives equal weight to each country. Bangladesh, Myanmar, Nepal, Sir Lanka and Timor-Leste have a body mass index target of less than 23 kg/m2. Total cholesterol targets were set at less than 5.0 mmol/L (190 mg/dL) for patients without a history of CVD and less than 4.0 mmol/L (152 mg/dL) for patients with a history of CVD.

Lifestyle targets

	Prevalence (%)	Estimated average marginal effect (95% CI)	*P* values	Risk ratio (95% CI)	*P* values
**Not currently smoking**					
**Age, years**					
25-44	81.4 (77.6 to 84.6)	0 (ref)		1 (ref)	
45-54	83.6 (80.6 to 86.3)	1.75 (-3.08, 6.57)	0.478	1.01 (0.98, 1.04)	0.479
55-69	81.6 (78.5 to 82.3)	7.35 (2.59, 12.11)	0.002	1.04 (1.01, 1.07)	0.003
**Sex**					
Male	66.1 (62.9 to 69.2)	0 (ref)		1 (ref)	
Female	96.8 (95.8 to 97.5)	21.50 (18.46, 24.54)	<0.0001	1.12 (1.11, 1.14)	<0.0001
**Education**					
No schooling	86.0 (82.7 to 88.8)	0 (ref)		1 (ref)	
Primary	80.4 (77.6 to 82.9)	-4.17 (-9.91, 1.57)	0.155	0.98 (0.95, 1.01)	0.151
Secondary or higher	83.8 (81.2 to 86.2)	-5.88 (-12.44, 0.69)	0.079	0.97 (0.94, 1.00)	0.077
**Not currently drinking**					
**Age, years**					
25-44	80.4 (76.1 to 84.0)	0 (ref)		1 (ref)	
45-54	83.2 (79.8 to 86.1)	5.44 (-0.15, 11.03)	0.057	1.03 (1.00, 1.06)	0.059

55-70	85.6 (83.0 to 87.8)	10.37 (4.95, 15.79)	<0.0001	1.06 (1.03, 1.09)	<0.0001
**Sex**					
Male	72.7 (69.2 to 75.9)	0 (ref)		1 (ref)	
Female	91.5 (89.9 to 92.8)	18.57 (15.27, 21.88)	<0.0001	1.11 (1.09, 1.13)	<0.0001
**Education**					
No schooling	87.3 (83.4 to 90.4)	0 (ref)		1 (ref)	
Primary	85.9 (83.3 to 88.2)	-3.28 (-8.51, 1.95)	0.218	0.98 (0.95, 1.01)	0.217
Secondary or higher	77.0 (73.9 to 79.9)	-7.96 (-12.97, -2.96)	0.002	0.96 (0.93, 0.98)	0.002
**Sufficient physical activity**					
**Age, years**					
25-44	76.0 (72.1 to 79.4)	0 (ref)		1 (ref)	
45-54	66.8 (62.8 to 70.6)	-4.43 (-10.18, 1.32)	0.131	0.93 (0.84, 1.02)	0.126
55-70	43.7 (40.0 to 47.5)	-14.92 (-20.48, -9.37)	<0.0001	0.76 (0.69, 0.84)	<0.0001
**Sex**					
Male	65.0 (61.8 to 68.1)	0 (ref)		1 (ref)	
Female	65.8 (62.7 to 68.8)	-5.81 (-9.77, -1.85)	0.004	0.90 (0.83, 0.97)	0.004
**Education**					
No schooling	69.6 (64.9 to 74.0)	0 (ref)		1 (ref)	
Primary	65.3 (61.7 to 68.8)	-3.81 (-12.01, 4.38)	0.362	0.93 (0.80, 1.08)	0.352
Secondary or higher	62.6 (59.1 to 65.9)	-0.32 (-9.02, 8.39)	0.943	0.99 (0.85, 1.16)	0.943
**Dietary fruit and vegetables ≥5 servings/day**					
**Age, years**					
25-44	14.4 (11.5 to 17.7)	0 (ref)		1 (ref)	
45-54	15.8 (13.1 to 18.9)	0.26 (-5.02, 5.54)	0.922	1.01 (0.76, 1.36)	0.922
55-70	20.4 (17.6 to 23.5)	2.94 (-2.38, 8.25)	0.278	1.16 (0.88, 1.55)	0.298
**Sex**					

Male	17.2 (14.7 to 20.1)	0 (ref)		1 (ref)	
Female	15.4 (13.4 to 17.6)	-0.99 (-4.09, 2.12)	0.532	0.95 (0.81, 1.11)	0.529
**Education**					
No schooling	5.2 (3.9 to 7.1)	0 (ref)		1 (ref)	
Primary	13.3 (11.1 to 16.0)	2.31 (-3.30, 7.93)	0.419	1.14 (0.82, 1.60)	0.436
Secondary or higher	25.8 (22.7 to 29.1)	4.45 (-1.18, 10.08)	0.122	1.27 (0.91, 1.78)	0.157

Prevalence has been recalculated based on the adjusted weight. This gives equal weight to each country.

Supplementary Table S9: Association between individual characteristics and guideline-recommended treatment, metabolic and lifestyle targets in people with diabetes (Multivariable Poisson regression model)

Treatment targets

	Prevalence (%)	Estimated average marginal effect (95% CI)	*P* values	Risk ratio (95% CI)	*P* values
**Glucose-lowering drug**					
**Age, years**					
25-44	37.41 (30.78, 44.56)	0 (ref)		1 (ref)	
45-54	57.95 (54.29, 61.51)	18.93 (11.74, 26.11)	<0.0001	1.48 (1.25, 1.77)	<0.0001
55-69	65.46 (62.23, 68.56)	25.03 (17.96, 32.09)	<0.0001	1.64 (1.38, 1.95)	<0.0001
**Sex**					
Male	58.98 (55.02, 62.83)	0 (ref)		1 (ref)	
Female	56.94 (53.90, 59.93)	-0.34 (-4.79, 4.11)	0.881	0.99 (0.92, 1.07)	0.881
**Education**					
No schooling	51.70 (44.72, 58.61)	0 (ref)		1 (ref)	
Primary	60.21 (55.79, 64.46)	12.76 (5.00, 20.53)	0.001	1.26 (1.08, 1.47)	0.003
Secondary or higher	57.16 (53.90, 60.36)	8.69 (0.70, 16.67)	0.033	1.18 (1.01, 1.38)	0.042

**Statins**					
**Age, years**					
40-54	8.10 (6.51, 10.04)	0 (ref)		1 (ref)	
55-70	13.85 (12.02, 15.92)	4.47 (2.11, 6.82)	<0.0001	1.52 (1.21, 1.93)	<0.0001
**Sex**					
Male	11.37 (9.45, 13.62)	0 (ref)		1 (ref)	
Female	10.96 (9.40, 12.75)	0.74 (-1.51, 2.99)	0.519	1.07 (0.87, 1.32)	0.523
**Education**					
No schooling	10.22 (6.73, 15.24)	0 (ref)		1 (ref)	
Primary	10.07 (8.24, 12.26)	0.69 (-4.02, 5.40)	0.774	1.07 (0.65, 1.76)	0.778
Secondary or higher	11.84 (10.01, 13.95)	2.89 (-1.30, 7.09)	0.176	1.31 (0.85, 2.02)	0.223
**Antihypertensive drug**					
**Age, years**					
25-44	16.11 (11.78, 21.65)	0 (ref)		1 (ref)	
45-54	34.49 (31.46, 37.66)	22.47 (13.92, 31.02)	<0.0001	1.77 (1.34, 2.34)	<0.0001
55-70	52.83 (49.66, 55.98)	35.01 (26.34, 43.67)	<0.0001	2.20 (1.66, 2.91)	<0.0001
**Sex**					
Male	33.51 (30.32, 36.86)	0 (ref)		1 (ref)	
Female	43.68 (40.92, 46.48)	12.84 (8.10, 17.58)	<0.0001	1.27 (1.16, 1.39)	<0.0001
**Education**					
No schooling	38.24 (32.53, 44.30)	0 (ref)		1 (ref)	
Primary	37.29 (33.59, 41.14)	14.61 (6.16, 23.07)	0.001	1.35 (1.12, 1.62)	0.002
Secondary or higher	40.49 (37.54, 43.51)	14.84 (5.45, 24.24)	0.002	1.35 (1.10, 1.66)	0.004
**Aspirin**					
**Age, years**					
25-44	9.34 (3.72, 21.55)	0 (ref)		1 (ref)	

45-54	25.94 (20.26, 32.56)	13.96 (5.29, 22.62)	0.002	2.18 (1.13, 4.19)	0.020
55-70	29.18 (24.42, 34.44)	17.19 (8.85, 25.52)	<0.0001	2.45 (1.27, 4.71)	0.007
**Sex**					
Male	23.52 (18.91, 28.85)	0 (ref)		1 (ref)	
Female	29.55 (24.21, 35.53)	5.34 (-0.35, 11.03)	0.066	1.22 (0.98, 1.52)	0.070
**Education**					
No schooling	29.28 (16.05, 47.29)	0 (ref)		1 (ref)	
Primary	21.79 (16.75, 27.84)	10.48 (1.73, 19.24)	0.019	1.57 (1.05, 2.35)	0.028
Secondary or higher	31.13 (26.77, 35.87)	11.15 (2.72, 19.57)	0.010	1.60 (1.07, 2.40)	0.022

Prevalence has been recalculated based on the adjusted weight. This gives equal weight to each country. The multivariable Poisson regression models have been adjusted for age group, sex and education, with the inclusion of country-level fixed effects.

Metabolic targets

	Prevalence (%)	Estimated average marginal effect (95% CI)	*P* values	Risk ratio (95% CI)	*P* values
**Fasting blood-glucose <6.1 mmol/L**					
**Age, years**					
25-44	63.10 (56.70, 69.07)	0 (ref)		1 (ref)	
45-54	39.39 (36.06, 42.82)	-19.59 (-26.26, -12.93)	<0.0001	0.67 (0.59, 0.76)	<0.0001
55-69	39.28 (36.01, 42.65)	-20.33 (-27.14, -13.52)	<0.0001	0.66 (0.58, 0.75)	<0.0001
**Sex**					
Male	43.68 (39.91, 47.52)	0 (ref)		1 (ref)	
Female	43.65 (40.51, 46.86)	-0.11 (-4.30, 4.08)	0.959	1.00 (0.91, 1.10)	0.959
**Education**					
No schooling	47.05 (40.71, 53.49)	0 (ref)		1 (ref)	
Primary	42.55 (37.73, 47.51)	-9.45 (-17.06, -1.83)	0.015	0.81 (0.69, 0.95)	0.011

Secondary or higher	43.35 (40.46, 46.29)	-6.32 (-13.96, 1.32)	0.105	0.87 (0.75, 1.02)	0.092
**Body mass index <25 kg/m²**					
**Age, years**					
25-44	40.69 (33.58, 48.21)	0 (ref)		1 (ref)	
45-54	23.68 (20.77, 26.86)	-10.80 (-17.03, -4.57)	0.001	0.70 (0.57, 0.85)	<0.0001
55-70	24.76 (21.79, 27.98)	-10.71 (-16.88, -4.54)	0.001	0.70 (0.58, 0.85)	<0.0001
**Sex**					
Male	34.03 (30.16, 38.12)	0 (ref)		1 (ref)	
Female	21.64 (19.20, 24.30)	-12.05 (-16.11, -7.99)	<0.0001	0.64 (0.56, 0.74)	<0.0001
**Education**					
No schooling	35.15 (29.03, 41.80)	0 (ref)		1 (ref)	
Primary	28.09 (23.39, 33.32)	-12.30 (-20.33, -4.27)	0.003	0.69 (0.55, 0.86)	0.001
Secondary or higher	24.36 (21.78, 27.14)	-14.97 (-23.37, -6.57)	<0.0001	0.62 (0.49, 0.78)	<0.0001
**Blood pressure <140/90 mm Hg**					
**Age, years**					
25-44	68.35 (62.35, 73.80)	0 (ref)		1 (ref)	
45-54	50.97 (47.57, 54.37)	-13.93 (-20.33, -7.54)	<0.0001	0.79 (0.71, 0.88)	<0.0001
55-70	38.46 (35.51, 41.49)	-26.75 (-32.95, -20.54)	<0.0001	0.59 (0.53, 0.66)	<0.0001
**Sex**					
Male	49.79 (46.17, 53.41)	0 (ref)		1 (ref)	
Female	47.84 (45.27, 50.42)	-1.45 (-5.55, 2.64)	0.486	0.97 (0.89, 1.06)	0.485
**Education**					
No schooling	48.32 (41.89, 54.81)	0 (ref)		1 (ref)	
Primary	52.49 (48.53, 56.42)	-1.67 (-9.72, 6.38)	0.684	0.97 (0.82, 1.14)	0.681
Secondary or higher	46.00 (43.10, 48.93)	-2.30 (-10.68, 6.09)	0.592	0.95 (0.81, 1.13)	0.587
**Total cholesterol <5.0 mmol/L**					

**Age, years**					
25-44	63.97 (57.60, 69.89)	0 (ref)		1 (ref)	
45-54	61.88 (58.42, 65.21)	0.29 (-5.32, 5.90)	0.919	1.00 (0.92, 1.10)	0.920
55-70	54.91 (51.79, 57.99)	-7.62 (-13.36, -1.89)	0.009	0.88 (0.80, 0.97)	0.007
**Sex**					
Male	66.50 (63.11, 69.73)	0 (ref)		1 (ref)	
Female	53.24 (50.42, 56.04)	-11.93 (-15.77, -8.09)	<0.0001	0.82 (0.77, 0.87)	<0.0001
**Education**					
No schooling	63.29 (56.60, 69.50)	0 (ref)		1 (ref)	
Primary	58.91 (54.58, 63.11)	-1.60 (-8.72, 5.52)	0.660	0.97 (0.87, 1.09)	0.657
Secondary or higher	58.73 (55.78, 61.61)	-3.52 (-11.12, 4.08)	0.364	0.94 (0.83, 1.07)	0.356

Prevalence has been recalculated based on the adjusted weight. This gives equal weight to each country. The multivariable Poisson regression models have been adjusted for age group, sex and education, with the inclusion of country-level fixed effects.

Lifestyle targets

	Prevalence (%)	Estimated average marginal effect (95% CI)	*P* values	Risk ratio (95% CI)	*P* values
**Not currently smoking**					
**Age, years**					
25-44	81.4 (77.6 to 84.6)	0 (ref)		1 (ref)	
45-54	83.6 (80.6 to 86.3)	0.21 (-4.44, 4.87)	0.928	1.00 (0.98, 1.03)	0.928
55-69	81.6 (78.5 to 82.3)	6.86 (2.16, 11.56)	0.004	1.04 (1.01, 1.07)	0.005
**Sex**					
Male	66.1 (62.9 to 69.2)	0 (ref)		1 (ref)	
Female	96.8 (95.8 to 97.5)	22.00 (18.75, 25.25)	<0.0001	1.13 (1.11, 1.15)	<0.0001
**Education**					

No schooling	86.0 (82.7 to 88.8)	0 (ref)		1 (ref)	
Primary	80.4 (77.6 to 82.9)	0.92 (-4.36, 6.19)	0.734	1.01 (0.98, 1.03)	0.734
Secondary or higher	83.8 (81.2 to 86.2)	2.25 (-4.08, 8.58)	0.487	1.01 (0.98, 1.05)	0.488
**Not currently drinking**					
**Age, years**					
25-44	80.4 (76.1 to 84.0)	0 (ref)		1 (ref)	
45-54	83.2 (79.8 to 86.1)	3.45 (-2.06, 8.96)	0.219	1.02 (0.99, 1.05)	0.222
55-70	85.6 (83.0 to 87.8)	9.05 (3.47, 14.62)	0.001	1.05 (1.02, 1.09)	0.002
**Sex**					
Male	72.7 (69.2 to 75.9)	0 (ref)		1 (ref)	
Female	91.5 (89.9 to 92.8)	18.16 (14.79, 21.53)	<0.0001	1.11 (1.09, 1.13)	<0.0001
**Education**					
No schooling	87.3 (83.4 to 90.4)	0 (ref)		1 (ref)	
Primary	85.9 (83.3 to 88.2)	1.34 (-3.94, 6.63)	0.619	1.01 (0.98, 1.04)	0.619
Secondary or higher	77.0 (73.9 to 79.9)	-0.64 (-5.89, 4.61)	0.810	1.00 (0.97, 1.03)	0.810
**Sufficient physical activity**					
**Age, years**					
25-44	76.0 (72.1 to 79.4)	0 (ref)		1 (ref)	
45-54	66.8 (62.8 to 70.6)	-4.62 (-10.48, 1.23)	0.122	0.93 (0.84, 1.02)	0.117
55-70	43.7 (40.0 to 47.5)	-15.30 (-20.97, -9.62)	<0.0001	0.75 (0.68, 0.83)	<0.0001
**Sex**					
Male	65.0 (61.8 to 68.1)	0 (ref)		1 (ref)	
Female	65.8 (62.7 to 68.8)	-5.94 (-10.11, -1.77)	0.005	0.90 (0.83, 0.97)	0.005
**Education**					
No schooling	69.6 (64.9 to 74.0)	0 (ref)		1 (ref)	
Primary	65.3 (61.7 to 68.8)	-7.08 (-15.61, 1.44)	0.103	0.88 (0.76, 1.02)	0.091

Secondary or higher	62.6 (59.1 to 65.9)	-4.92 (-14.02, 4.18)	0.289	0.92 (0.78, 1.07)	0.278
**Dietary fruit and vegetables ≥5 servings/day**					
**Age, years**					
25-44	14.4 (11.5 to 17.7)	0 (ref)		1 (ref)	
45-54	15.8 (13.1 to 18.9)	0.29 (-5.02, 5.60)	0.915	1.02 (0.76, 1.36)	0.915
55-70	20.4 (17.6 to 23.5)	2.81 (-2.58, 8.21)	0.306	1.16 (0.87, 1.54)	0.325
**Sex**					
Male	17.2 (14.7 to 20.1)	0 (ref)		1 (ref)	
Female	15.4 (13.4 to 17.6)	-0.92 (-4.18, 2.34)	0.581	0.95 (0.81, 1.13)	0.579
**Education**					
No schooling	5.2 (3.9 to 7.1)	0 (ref)		1 (ref)	
Primary	13.3 (11.1 to 16.0)	2.56 (-2.95, 8.07)	0.363	1.16 (0.83, 1.62)	0.384
Secondary or higher	25.8 (22.7 to 29.1)	4.59 (-1.09, 10.27)	0.113	1.29 (0.91, 1.81)	0.148

Prevalence has been recalculated based on the adjusted weight. This gives equal weight to each country. The multivariable Poisson regression models have been adjusted for age group, sex and education, with the inclusion of country-level fixed effects.

Appendix 6: Sensitivity analysis

Supplementary Table S10: Prevalence of treatment, metabolic and lifestyle targets for CVD prevention in patients with diabetes, by income group

	Prevalence (95% CI)			
	Overall	Upper-Middle Income Countries	Lower-Middle Income Countries	Low-Income Countries
**A. Treatment targets**				
Glucose-lowering drug	64.52 (61.59, 67.35)	71.85 (68.88, 74.64)	63.39 (59.74, 66.89)	59.49 (49.84, 68.47)
Statins	10.96 (9.17, 13.05)	17.73 (15.30, 20.44)	9.44 (7.16, 12.35)	7.88 (3.78, 15.72)
Antihypertensive drug	51.70 (47.93, 55.45)	61.59 (58.11, 64.95)	52.41 (47.76, 57.02)	40.02 (29.96, 50.99)

Aspirin	23.24 (19.12, 27.94)	35.63 (30.99, 40.55)	20.07 (15.13, 26.12)	20.17 (10.38, 35.51)
**B. Metabolic targets**				
Fasting blood glucose <6.1 mmol/L	39.69 (36.74, 42.71)	36.99 (33.84, 40.26)	37.87 (34.19, 41.69)	47.48 (38.45, 56.68)
Body mass index <25 kg/m²	31.28 (28.55, 34.15)	17.00 (14.75, 19.51)	30.32 (27.21, 33.64)	47.95 (39.15, 56.89)
Blood pressure <140/90 mm Hg	50.90 (48.14, 53.65)	50.67 (47.65, 53.68)	51.50 (48.12, 54.86)	49.44 (40.75, 58.16)
Total cholesterol <5.0 mmol/L	63.87 (60.93, 66.72)	56.29 (52.91, 59.61)	63.06 (59.04, 66.92)	73.58 (65.97, 80.00)
**C. Lifestyle targets**				
Not currently smoking	87.20 (85.10, 89.03)	83.46 (80.73, 85.87)	85.57 (82.38, 88.26)	95.45 (91.16, 97.72)
Not currently drinking	88.65 (86.47, 90.52)	89.88 (88.08, 91.43)	91.39 (89.33, 93.09)	79.68 (70.77, 86.40)
Sufficient physical activity	54.66 (51.82, 57.47)	36.46 (33.40, 39.65)	57.42 (53.73, 61.03)	64.59 (56.93, 71.57)
Dietary fruit and vegetables ≥5 servings/day	16.92 (14.67, 19.44)	20.31 (17.51, 23.43)	17.36 (14.48, 20.67)	12.37 (7.02, 20.88)

Prevalence was recalculated based on weights provided by individual surveys and the proportion of each country's population aged 25-69. The prevalence of statin use was assessed only in people with diabetes aged 40 years or older. The prevalence of aspirin use was assessed in people with diabetes with a history of CVD and a 10-year risk of CVD greater than 20%, as calculated using the WHO CVD Risk Laboratory-based Chart. Bangladesh, Myanmar, Nepal, Sir Lanka and Timor-Leste have a body mass index target of less than 23 kg/m2. Total cholesterol targets were set at less than 5.0 mmol/L (190 mg/dL) for patients without a history of CVD and less than 4.0 mmol/L (152 mg/dL) for patients with a history of CVD.

Supplementary Table S11: Prevalence of treatment, metabolic and lifestyle targets for CVD prevention in patients with diabetes, by region

Treatment targets

Region	Prevalence (95% CI)				
	Glucose-lowering drug	Statins	Antihypertensive drug	Aspirin	All 4 targets met
Africa	58.50 (51.16, 65.47)	9.17 (6.05, 13.67)	35.93 (28.75, 43.79)	16.59 (9.80, 26.70)	9.40 (6.22, 13.97)
Americas	51.50 (44.32, 58.62)	9.67 (5.81, 15.65)	57.87 (48.76, 66.48)	16.38 (7.38, 32.48)	6.48 (3.66, 11.22)
Western Pacific	27.36 (22.19, 33.23)	8.92 (5.79, 13.49)	60.82 (52.78, 68.31)	30.31 (22.71, 39.17)	5.20 (3.23, 8.28)
European	59.50 (55.67, 63.22)	11.11 (8.68, 14.12)	61.95 (57.65, 66.06)	35.40 (31.05, 40.01)	7.36 (5.56, 9.69)
Eastern Mediterranean	59.13 (55.34, 62.80)	12.38 (10.17, 14.98)	58.99 (54.67, 63.19)	28.67 (23.04, 35.04)	11.72 (9.24, 14.77)
South-East Asia	78.33 (73.21, 82.71)	11.55 (8.04, 16.31)	57.41 (50.76, 63.81)	22.87 (15.67, 32.12)	9.19 (6.61, 12.64)

Prevalence was recalculated based on weights provided by individual surveys and the proportion of each country's population aged 25-69.

Metabolic targets

Region	Prevalence (95% CI)				
	Body mass index <25 kg/m²	Blood pressure <140/90 mm Hg	Total cholesterol <5.0 mmol/L	Fasting blood-glucose <6.1 mmol/L	All 4 targets met
Africa	46.94 (39.79, 54.22)	43.16 (36.53, 50.04)	50.48 (43.73, 57.21)	76.81 (71.27, 81.57)	15.31 (10.71, 21.43)
Americas	51.42 (44.19, 58.59)	23.08 (17.53, 29.74)	77.79 (71.64, 82.92)	56.68 (49.59, 63.51)	7.12 (4.11, 12.07)
Western Pacific	44.22 (38.38, 50.23)	22.92 (18.50, 28.04)	64.60 (58.73, 70.07)	52.37 (46.14, 58.54)	8.50 (5.65, 12.59)
European	38.37 (34.84, 42.02)	13.56 (11.17, 16.37)	31.44 (28.13, 34.94)	48.50 (44.63, 52.39)	2.21 (1.43, 3.39)
Eastern Mediterranean	34.73 (31.32, 38.32)	21.65 (18.84, 24.75)	45.67 (41.80, 49.58)	63.48 (59.65, 67.15)	4.38 (3.02, 6.32)
South-East Asia	35.94 (31.02, 41.18)	32.17 (27.81, 36.87)	57.26 (52.76, 61.65)	58.10 (52.39, 63.61)	7.53 (5.08, 11.03)

Prevalence was recalculated based on weights provided by individual surveys and the proportion of each country's population aged 25-69.

Lifestyle targets

Region	Prevalence (95% CI)				
	Not currently smoking	Not currently drinking	Sufficient physical	Fruit and vegetables	All 4 targets met

			activity	≥5 servings/day	
Africa	93.56 (89.74, 96.02)	82.28 (75.49, 87.51)	63.44 (57.19, 69.27)	14.57 (10.07, 20.62)	9.16 (5.31, 15.36)
Americas	85.02 (78.60, 89.76)	63.89 (56.93, 70.32)	59.24 (52.67, 65.49)	6.25 (3.41, 11.16)	0.27 (0.06, 1.19)
Western Pacific	70.63 (64.39, 76.19)	62.06 (55.86, 67.90)	58.80 (52.72, 64.63)	13.58 (10.02, 18.16)	5.74 (3.50, 9.26)
European	85.50 (82.49, 88.06)	71.39 (67.87, 74.67)	46.51 (42.60, 50.46)	35.59 (31.95, 39.41)	13.08 (10.64, 15.97)
Eastern Mediterranean	86.48 (83.78, 88.78)	99.63 (99.25, 99.82)	38.00 (34.55, 41.58)	14.10 (12.07, 16.41)	4.03 (3.04, 5.33)
South-East Asia	83.20 (78.43, 87.09)	94.41 (91.95, 96.15)	58.46 (53.24, 63.50)	16.30 (12.23, 21.40)	6.92 (4.95, 9.60)

Prevalence was recalculated based on weights provided by individual surveys and the proportion of each country's population aged 25-69.

Supplementary Table S12: Prevalence of guideline-recommended targets for all components of treatment, metabolic and lifestyle in people with diabetes by World Bank income group and region

By World Bank income group

	Prevalence (95% CI)			
	Overall	Upper-Middle Income Countries	Lower-Middle Income Countries	Low-Income Countries
Treatment targets all met	9.51 (7.94, 11.35)	13.97 (11.96, 16.24)	7.44 (5.73, 9.61)	11.03 (6.51, 18.07)
Metabolic targets all met	8.73 (6.93, 10.93)	3.10 (2.11, 4.52)	7.66 (5.78, 10.08)	17.26 (11.22, 25.63)
Lifestyle targets all met	7.43 (5.86, 9.39)	6.22 (4.76, 8.08)	7.47 (5.86, 9.48)	8.51 (3.88, 17.62)

Prevalence was recalculated based on weights provided by individual surveys and the proportion of each country's population aged 25-69.

By region

	Prevalence (95% CI)					
	Africa	Americas	Western Pacific	Europe	Eastern Mediterranean	South-East Asia
Treatment targets all met	9.40 (6.22, 13.97)	6.48 (3.66, 11.22)	5.20 (3.23, 8.28)	7.36 (5.56, 9.69)	11.72 (9.24, 14.77)	9.19 (6.61, 12.64)

Metabolic targets all met	15.31 (10.71, 21.43)	7.12 (4.11, 12.07)	8.50 (5.65, 12.59)	2.21 (1.43, 3.39)	4.38 (3.02, 6.32)	7.53 (5.08, 11.03)
Lifestyle targets all met	9.16 (5.31, 15.36)	0.27 (0.06, 1.19)	5.74 (3.50, 9.26)	13.08 (10.64, 15.97)	4.03 (3.04, 5.33)	6.92 (4.95, 9.60)

Prevalence was recalculated based on weights provided by individual surveys and the proportion of each country's population aged 25-69.

Supplementary Table S13: Individual characteristics of the diabetes, high risk of CVD and established CVD group

	Diabetes (N=6789)		10 years CVD risk >30% (N=608)		10 years CVD risk >30% (N=485)	
Characteristics	Unweighted, n	Weighted, %	Unweighted, n	Weighted, %	Unweighted, n	Weighted, %
**Age**						
25-39 years	878	18.2	1	0.4	2	0.5
40-54 years	2610	38.5	48	2.4	59	3.6
55-69 years	3301	43.3	559	14.7	424	12.9
**Sex**						
Male	2479	45.2	309	10.9	173	7.1
Female	4310	54.8	299	4.4	312	7.0
**Country economic status**						
Upper-middle	2260	28.9	31	3.8	37	6.4
Lower-middle	3992	55.3	304	6.6	267	7.2
Low	537	15.8	273	10.7	181	7.2
**Region**						
Africa	889	28.9	26	2.0	43	5.8
Americas	367	5.3	0	0	2	3.6
Western Pacific	713	15.8	46	2.6	35	5.8

European	1398	21.1	365	2.3	192	11.9
Eastern Mediterranean	1853	15.8	168	8.6	152	6.8
South-East Asia	1569	13.2	3	0.3	61	5.5
**Education**						
No schooling	951	11.9	56	3.8	76	9.4
Primary	2493	38.1	638	3．8	144	5.9
Secondary or higher	3128	50.0	844	10.7	240	7.3
**Area of residence**						
Urban	2599	56.2	251	7.9	182	6.7
Rural	1607	43.8	162	7.3	133	7.1

CVD=cardiovascular. Prevalence was recalculated based on the adjusted weight, giving equal weight to each country.

Supplementary Table S14: Prevalence of aspirin use in WHO ISH risk > 30% subgroup, by income group and region

	Prevalence (95% CI)			
	Overall	Upper-Middle Income Countries	Lower-Middle Income Countries	Low-Income Countries
Aspirin	35.62 (31.65, 39.80)	53.29 (46.21, 60.24)	23.60 (19.81, 27.85)	41.84 (28.06, 57.03)
Treatment targets all met	6.27 (5.38, 7.30)	12.54 (10.20, 15.31)	3.66 (2.98, 4.50)	3.91 (1.83, 8.16)

Prevalence was recalculated based on the adjusted weight, giving equal weight to each country.

By region

	Prevalence (95% CI)					
	Africa	Americas	Western Pacific	Europe	Eastern Mediterranean	South-East Asia

Aspirin	26.17 (16.41, 39.03)	23.88 (11.54, 43.00)	18.94 (13.52, 25.89)	44.19 (38.12, 50.43)	60.89 (52.51, 68.68)	18.82 (13.49, 25.63)
Treatment targets all met	4.32 (2.89, 6.43)	4.94 (2.65, 9.02)	3.35 (1.98, 5.62)	6.07 (4.12, 8.87)	14.56 (11.68, 18.02)	4.96 (3.76, 6.52)

Prevalence was recalculated based on the adjusted weight, giving equal weight to each country.

Supplementary Table S15: Prevalence of blood pressure < 130/80 mm Hg, by income group and region

	Prevalence (95% CI)			
	Overall	Upper-Middle Income Countries	Lower-Middle Income Countries	Low-Income Countries
Blood pressure <130/80 mm Hg	24.07 (22.06, 26.21)	24.09 (21.34, 27.06)	23.82 (21.45, 26.37)	24.95 (17.41, 34.39)
All metabolic target met	5.70 (4.25, 7.60)	2.13 (1.39, 3.23)	6.27 (4.65, 8.41)	10.23 (4.68, 20.92)

Prevalence was recalculated based on the adjusted weight, giving equal weight to each country.

By region

	Prevalence (95% CI)					
	Africa	Americas	Western Pacific	Europe	Eastern Mediterranean	South-East Asia
Blood pressure <130/80 mm Hg	26.40 (21.46, 32.01)	44.03 (36.90, 51.43)	31.14 (26.83, 35.80)	11.59 (9.40, 14.21)	20.01 (16.94, 23.47)	27.36 (21.71, 33.85)
All metabolic target met	8.07 (4.55, 13.92)	5.33 (3.05, 9.14)	5.22 (3.09, 8.71)	1.35 (0.68, 2.65)	2.45 (1.60, 3.72)	12.06 (7.15, 19.63)

Prevalence was recalculated based on the adjusted weight, giving equal weight to each country.

Appendix 7: STROBE Checklist

Checklist of items that should be included in reports of cross-sectional studies

	Item No	Recommendation	Item No
**Title and abstract**	1	(*a*) Indicate the study's design with a commonly used term in the title or the abstract	1
		(*b*) Provide in the abstract an informative and balanced summary of what was done and what was found	2, 3
**Introduction**			
Background/rationale	2	Explain the scientific background and rationale for the investigation being reported	3, 4
Objectives	3	State specific objectives, including any prespecified hypotheses	3, 4
**Methods**			
Study design	4	Present key elements of study design early in the paper	5
Setting	5	Describe the setting, locations, and relevant dates, including periods of recruitment, exposure, follow-up, and data collection	5
Participants	6	(*a*) Give the eligibility criteria, and the sources and methods of selection of participants	5
Variables	7	Clearly define all outcomes, exposures, predictors, potential confounders, and effect modifiers. Give diagnostic criteria, if applicable	5, 6
Data sources/ measurement	8*	For each variable of interest, give sources of data and details of methods of assessment (measurement). Describe comparability of assessment methods if there is more than one group	5
Bias	9	Describe any efforts to address potential sources of bias	5
Study size	10	Explain how the study size was arrived at	8
Quantitative variables	11	Explain how quantitative variables were handled in the analyses. If applicable, describe which groupings were chosen and why	5, 6
Statistical methods	12	(*a*) Describe all statistical methods, including those used to control for confounding	6, 7
		(*b*) Describe any methods used to examine subgroups and interactions	7
		(*c*) Explain how missing data were addressed	7

		(*d*) If applicable, describe analytical methods taking account of sampling strategy	7
		(*e*) Describe any sensitivity analyses	7
**Results**			
Participants	13*	(a) Report numbers of individuals at each stage of study—eg numbers potentially eligible, examined for eligibility, confirmed eligible, included in the study, completing follow-up, and analysed	8
		(b) Give reasons for non-participation at each stage	8
		(c) Consider use of a flow diagram	8
Descriptive data	14*	(a) Give characteristics of study participants (eg demographic, clinical, social) and information on exposures and potential confounders	8, 9
		(b) Indicate number of participants with missing data for each variable of interest	8
Outcome data	15*	Report numbers of outcome events or summary measures	8, 9
Main results	16	(*a*) Give unadjusted estimates and, if applicable, confounder-adjusted estimates and their precision (eg, 95% confidence interval). Make clear which confounders were adjusted for and why they were included	8, 9, 10
		(*b*) Report category boundaries when continuous variables were categorized	8, 9, 10
		(*c*) If relevant, consider translating estimates of relative risk into absolute risk for a meaningful time period	8, 9, 10
Other analyses	17	Report other analyses done—eg analyses of subgroups and interactions, and sensitivity analyses	10
**Discussion**			
Key results	18	Summarise key results with reference to study objectives	10, 11
Limitations	19	Discuss limitations of the study, taking into account sources of potential bias or imprecision. Discuss both direction and magnitude of any potential bias	14
Interpretation	20	Give a cautious overall interpretation of results considering objectives, limitations, multiplicity of analyses, results from similar studies, and other relevant evidence	11, 12, 13, 14
Generalisability	21	Discuss the generalisability (external validity) of the study results	14

**Other information**			
Funding	22	Give the source of funding and the role of the funders for the present study and, if applicable, for the original study on which the present article is based	15

*Give information separately for exposed and unexposed groups.
